# Mitigation of Urban Heat Risks via Shading Stations: An Experimental Comparison of Fabric and Photovoltaic Shading

**DOI:** 10.1002/gch2.70134

**Published:** 2026-07-23

**Authors:** Liyue Jin, Xin Dong, Deo Prasad, Bao‐Jie He

**Affiliations:** ^1^ Centre for Climate–Resilient and Low–Carbon Cities School of Architecture and Urban Planning Key Laboratory of New Technology for Construction of Cities in Mountain Area Ministry of Education Chongqing University Chongqing China; ^2^ School of Civil Engineering and Architecture University of Jinan Jinan Shandong China; ^3^ School of Built Environment University of New South Wales Sydney NSW Australia; ^4^ School of Architecture, Design and Planning University of Queensland Brisbane QLD Australia

**Keywords:** fabric shading, microclimatic variables, outdoor thermal comfort, photovoltaic shading, thermal quality, weather condition

## Abstract

Exploring mitigation solutions is important for supporting heat‐resilient urban designs. Shading stations offer a practical means of blocking direct solar exposure and improving the thermal environment of the station. This study aimed to compare the heat mitigation performance of fabric and photovoltaic (PV) canopy shading in terms of microclimate regulation and outdoor thermal comfort improvement based on field experiments in subtropical Jiangsu, China. The results indicate that artificial shading could significantly reduce the universal thermal climate index (UTCI), with average daytime reductions of 3.5°C for fabric shading and 3.1°C for PV canopy shading. Although both types of shading stations were effective, they exhibited distinct mechanisms of action. Fabric shading provided more consistent cooling and radiation reduction at the ground level (0–0.5 m). During the full‐shading period (10:30–13:30), PV shading showed stronger downward longwave radiation, resulting in a weaker mitigation performance at pedestrian height (1.0–1.5 m) than that of fabric shading. Furthermore, the shading‐induced mitigation performance can be affected by underlying surfaces. Grass surfaces enhanced cooling via evapotranspiration, whereas high‐albedo hard surfaces sometimes contributed to heat accumulation. The differences in vertical temperatures verified the different mitigation mechanisms of the two types of shading stations along their height. Overall, this study provides a reference for designing artificial shading.

## Graphical abstract

PV shading relies primarily on solid panels to block direct solar radiation. As the transmittance of PV modules is close to zero, they effectively and consistently attenuate downward short‐wave radiation. With a surface reflectance of approximately 0.12, a portion of the short‐wave solar radiation is reflected, while the remaining energy is absorbed and converted into heat and electrical.

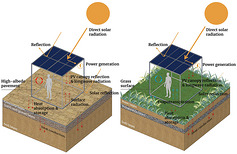

## 1 Introduction

Over several decades, global climate change has aggravated, leading to more frequent, severe, and intense extreme weather events, including extreme heat events, with significant environmental, social, and economic consequences. Associated with urbanization, the urban heat island (UHI) effect has become a critical heat‐related challenge that intensifies thermal discomfort, reduces work efficiency, and increases cooling energy demand [1]. As such, individuals conducting sporting and leisure activities outdoors often face stronger heat stress than ever before. Prolonged exposure to intense heat significantly increases the risk of heatstroke and other heat‐related illnesses, posing a substantial threat to public health [2]. Improving the thermal quality of public spaces, which is the basis of urban livability, is important to alleviate heat stress to secure public health and safety, sustain leisure and sporting activities, and ensure economic production. The development of effective heat mitigation and adaptation strategies is imperative to protect public health and well‐being, and ensure urban sustainability and resilience [3].

Outdoor thermal comfort (OTC) is an indicator of urban thermal quality (UTQ). The universal thermal climate index (UTCI) and physiological equivalent temperature (PET), which are primarily influenced by solar radiation, air temperature, wind speed, and humidity, are comprehensively linked to public health and well‐being. Therefore, mitigation strategies must focus on the regulation of OTC and associated variables to improve thermal quality. Various cooling approaches have been developed and examined, primarily in terms of urban morphology, blue‐green infrastructure (e.g., vegetation and water bodies), and innovative surface materials [4]. These interventions work in one or several ways to reduce solar exposure, enhance ventilation, lower radiation absorption, and cool ambient air [5].

Shading facilities are effective in reducing solar heat and regulating microclimatic variables [4]. Building shading stations in outdoor exposed spaces (e.g., streets, squares, parks, and rooftops) can reduce direct solar exposure and improve thermal environments. Prior studies have demonstrated the capacity of urban shading to alleviate heat stress [6], with most studies exploring the shading cast by trees and built forms (e.g., street canyons) in urban areas. Moreover, artificial shading components are frequently viewed as flexible alternatives or complements to trees and buildings; however, few empirical studies have directly compared the mitigation performance of different types of artificial shading facilities [7]. Typically, on hot and sunny days, shading devices can effectively block incoming solar radiation and reduce air temperatures beneath the shade, thus enhancing the OTC both physiologically and psychologically [8]. A case study in Port Said, Egypt showed that black fabric shading could lower predicted mean vote values by 22% and reduce PET by more than 4°C compared to unshaded spaces [9].

Decarbonization remains a fundamental strategy for addressing climate change [10]. In contrast to the excessive reliance on carbon‐intensive fuels, which has intensified the global energy crisis and accelerated greenhouse gas emissions, carbon‐free renewable energy has been promoted and implemented in cities [11]. Solar energy offers enormous potential for decarbonization, exhibiting various scenarios and wide application feasibility in on‐site and off‐site harvesting [12]. Urban buildings and their structural elements can serve as excellent platforms for solar energy utilization. Accordingly, a novel practice has been developed to integrate solar technologies with shading facilities, that is, photovoltaic (PV) canopy shading, to achieve both decarbonization and heat mitigation in public spaces [13]. It is regarded as a promising co‐benefit pathway for increasing decarbonization capacity while improving outdoor thermal environments. Some studies have explored PV‐induced impacts on microclimate and OTC, while most of them have modeled PV canopies on building roofs, facades, and walls. In comparison, limited studies have assumed the PV canopy as a street canopy and walking route, and there is a need to investigate the impacts of PV canopy shading on urban thermal quality. A good understanding of the heat mitigation performance of PV canopy shading will be conducive to the design of heat‐resilient walkable routes.

For PV canopy shading and fabric shading, the former can intercept incoming solar radiation and simultaneously generate electricity, whereas fabric shading can intercept solar radiation and ensure superior heat dissipation. It is essential to further clarify microclimate and OTC regulation performance to support decisions regarding design schemes [14]. Understanding how shading facilities interact with underlying surfaces (e.g., pervious surfaces, conventional hard surfaces, and reflective pavements) to intervene in the microclimate is also important for optimizing shading configurations. Empirical studies are necessary to reveal the thermal regulation effects of artificial shading.

Although some studies have investigated the cooling benefits of artificial shading facilities, they lack effective comparisons of the impacts of fabric and PV canopy shading on urban thermal quality based on empirical measurements. To address this gap, this study conducted a field experiment in Liyang City, Jiangsu Province, China, to quantitatively assess the urban thermal quality improvement by two common artificial shading types—PV canopy and black fabric—over two types of ground surface cover (e.g., grass and high‐albedo pavement) and under different weather conditions (e.g., cloudy hot days and sunny hot days) in hot seasons. Key climatic and OTC indicators, including *T_a_*, *T*_mrt_, and UTCI, were analyzed to explore the response to fabric and PV canopy shading. The findings provide empirical evidence and references for optimizing outdoor thermal environments by adopting shading stations.

## 2 Methodology

### 2.1 Study Area

The field experiment was conducted in an academic precinct in Liyang City, Jiangsu Province, China. According to the Köppen climate classification [15], the city has a humid subtropical monsoon climate (*Cwa*), characterized by hot and rainy summers, cold and dry winters, and significant annual temperature variations. Based on climate data from 1991 to 2020, the average air temperature in August is 28.2°C, with an extreme maximum daily temperature reaching up to 43.0°C. The average annual precipitation is 1198.8 mm, and the total annual sunshine duration ranges from 1400 to 2200 h.

**FIGURE 1:**
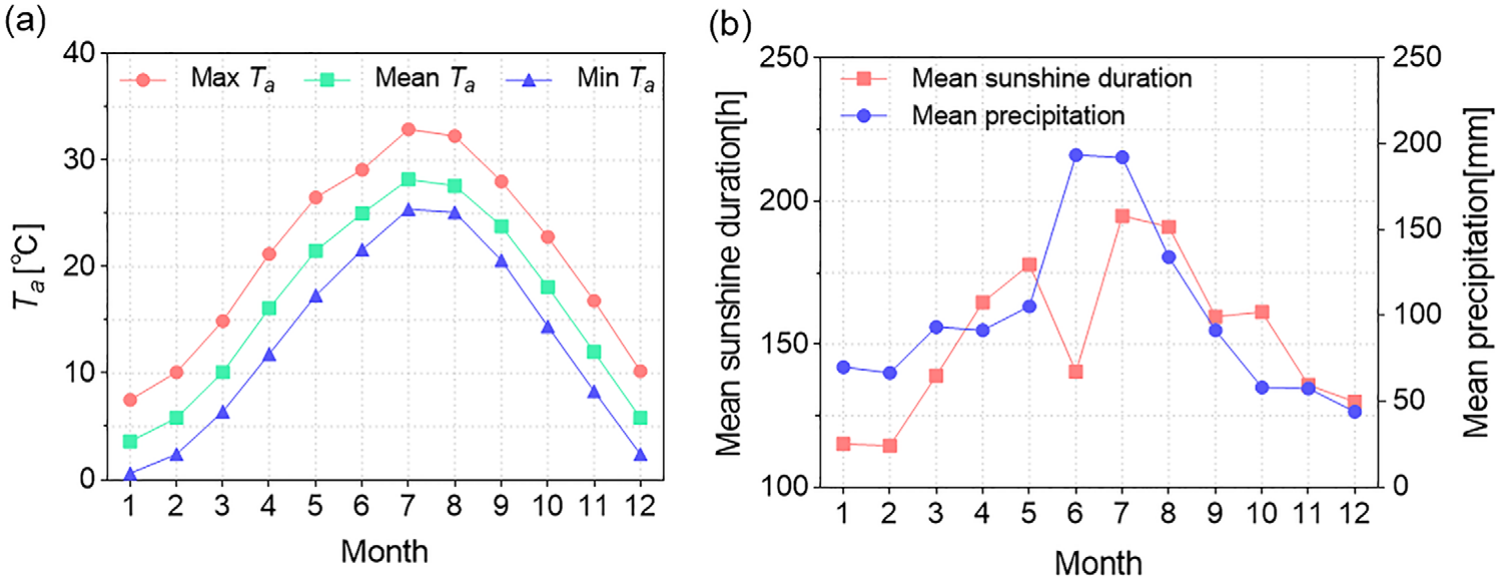
Monthly average, maximum, and minimum air temperature, sunshine duration, and precipitation in Liyang City, Jiangsu Province, China, from 1991 to 2020.

The period from June to September generally has high air temperatures and solar radiation inputs, with July and August being particularly hot (Figure 1). These months are associated with the most intense thermal stress and a strong need for mitigation and adaptation measures. Considering these climatic characteristics, field measurements were scheduled for August. The measurement period spanned three days, including one cloudy day (August 22nd) and two consecutive sunny days (August 23rd–24th) (Table 1).

**TABLE 1:** Combination of two shaded groups and ground surface cover for experimental investigation.

Shading facilities	Cloud condition	Heat condition	Date	Ground surface cover
PV & Fabric	Cloudy day	Hot day	2024.08.22: Scenario 1	Grass land surface
	Cloudless day	Extreme heat days	2024.08.23: Scenario 2	Grass land surface
			2024.08.24: Scenario 3	High‐albedo surface

### 2.2 Field Measurement

#### 2.2.1 Measurement location

Based on application contexts, artificial shading facilities are generally categorized into three types: walkway shading, plaza (courtyard) shading, and park shading. To better mimic real‐world conditions, we designed experimental scenarios to simulate the deployment of shading facilities that are commonly encountered during daily pedestrian movements in cities. We selected a microclimatic and thermal comfort field study site on the south side of an east‐west‐oriented street, with continuous three‐story buildings on the northern side. The site receives direct sunlight for most of the day, except during early morning and late afternoon.

#### 2.2.2 Measurement Scenarios: Shading Types, Underground Surface and Weather Conditions

**FIGURE 2:**
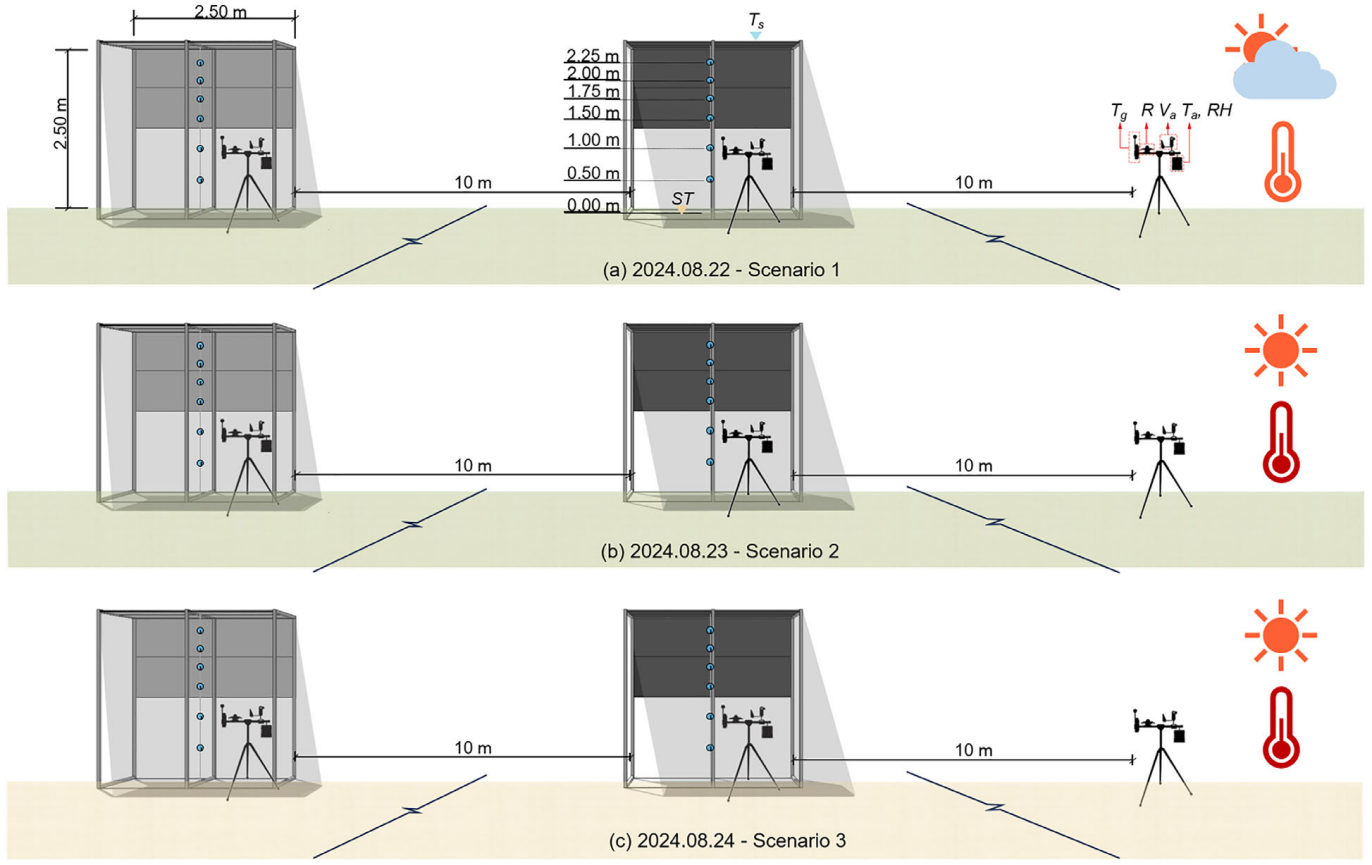
Field measurement duration, weather condition, and ground surface cover.

The two shading structures were designed with identical geometric dimensions of 1200 mm × 2400 mm × 2500 mm (length × width × height) (Figure 2). The top surface and upper half of the south‐facing side of each structure were covered with a canopy to provide shading protection for pedestrians’ heads and their upper bodies. The PV shading station consisted of eight monocrystalline silicon canopies with dimensions of 1200 mm × 550 mm × 3 mm, offering a rigid, opaque surface with high solar blocking efficiency and structural stability. The fabric shading facility was built using a black high‐density polyethylene (HDPE) mesh fabric, which is commonly used in cities for shading applications in China. The structure had a thickness of 0.3 mm and deployed size of 2400 mm × 2400 mm. The fabric offers partial solar blockage, good breathability, and diffuse‐light transmission. An unshaded control group was used for comparison purposes. Overall, these experimental settings enabled the analysis of how the shading design affected microclimatic and OTC performance.

Furthermore, two shaded groups were integrated with two types of ground surface cover to investigate the heat mitigation performance of the shading facilities (Table 1). To represent common urban environments, two types of ground surface cover were used: high‐albedo pavement (with an albedo of approximately 0.6–0.7) and a grass surface (albedo approximately 0.2–0.3). High‐albedo pavements, a widely used reflective hardscape material, are effective in reducing solar energy absorption through reflection; however, they may exacerbate human heat exposure owing to increased secondary shortwave and longwave radiation. The grass surface, characterized by lower reflectivity and higher evapotranspiration capacity, can mitigate near‐ground heat accumulation under extreme heat conditions, thereby improving the local microclimate.

Three field measurement scenarios were designed according to the weather conditions and ground surface cover (Figure 2). In each scenario, three groups were tested: PV canopy shading, black fabric shading, and direct solar exposure (DSE) control, representing two shaded groups and an unshaded control group. The two shaded groups were arranged with a 10‐m separation in the east‐west direction to avoid mutual shading during measurements. All three sites were equipped with the same sensors and layouts to ensure the consistency and comparability of the data.

#### 2.2.3 Measurement Period

To capture the temporal dynamics of the thermal environment, all measurements were segmented into four periods: A (midday/afternoon, 09:00–15:30), B (late afternoon, 15:30–18:35), C (night, 18:35–05:30) and D (early morning, 05:30–09:00). This classification was based on solar angles, diurnal heat load patterns, and conventional divisions in urban‐climate studies.

### 2.3 Microclimatic Variables and Sensors

**FIGURE 3:**
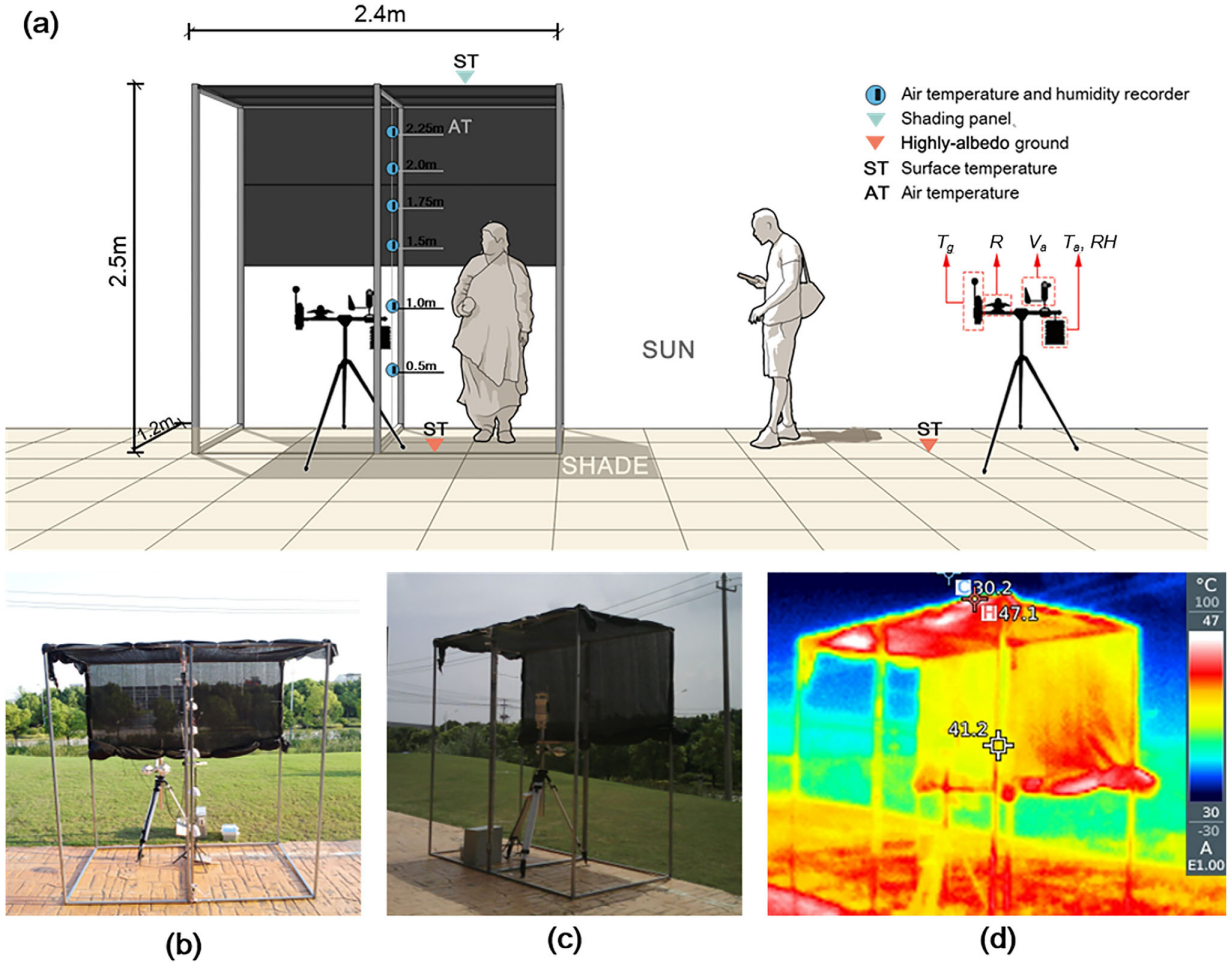
Experimental setup: (a) Schematic diagram of a fabric shading facilities with measuring devices and measurement points; visible images (b,c) and thermal image (d) of the underside of fabric shading facilities on high‐ albedo land surface. The colors and the values on the thermal image (d) indicate the surface temperature.

To comprehensively investigate the mitigation performance of shading facilities, this study measured a range of key microclimatic variables that can influence outdoor heat stress and thermal comfort of the occupants. These include air temperature (*T_a_*), black globe temperature (*T_g_*), surface temperature of the shading canopy (*T_s_*), ground surface temperature (*ST*), and background microclimatic variables. Dedicated instruments and sensors were deployed to capture horizontal and vertical environmental data under different shading scenarios. The following subsections describe the setup, measurement locations, and methodological concerns for each variable (Figure 3).

#### 2.3.1 Background Weather Measurement

At each measurement site, five key microclimatic variables were recorded: air temperature (*T_a_*, °C), relative humidity (*RH*, %), wind speed (*V_a_*, m/s), longwave and shortwave solar radiation (W/m^2^), and black globe temperature (*T_g_*, °C) (Table 2). The wind speed was measured at a height of 2 m above the ground using a Testo 425 anemometer. Air temperature, black globe temperature, relative humidity, and pyranometer were measured using a TRM‐ZS2 meteorological station, with sensors positioned at a height of 1.10 m above the ground, corresponding to the average center of gravity of an adult. All microclimatic variables were logged at 1‐min intervals. Surface temperatures were captured using a Testo‐865 thermal imager, with thermal images taken approximately every 30 min throughout the experiment.

**TABLE 2:** Details of meteorological sensors.

Parameter	Instrument	Sensor	Range	Accuracy
*T_a_*	Jinzhou Sunshine Weather station TRM‐ZS2‐BX	PTS‐3 air temperature sensor	−40 to 120°C	±0.2°C
*RH*		PTS‐3 humidity sensor	0–100%	±2%
Short and long wave radiation		TBB‐2 net all‐radiation meter/ Pyranometers	−2000 to 2000W/m^2^	±1W/m^2^
*T_g_*		PTWD‐4 black globe thermometer	−50 to 150°C	±0.2°C
*V_a_* (2m)	Testo 425	Thermal wire anemometer	0.01–30m/s	0.03m/s+4.0% (≤20m/s) 0.5m/s+5.0% (>20m/s)
*T_s_*/*ST*	Testo 175T‐3	NTC temperature sensor	−50 to 400°C(T‐type)	±0.5°C (−50 to 70°C)
*T_a_* (vertical)	Testo 174H	NTC temperature sensor	−20 to 70°C	±0.5°C (−20 to 70°C)

#### 2.3.2 *T_g_* Measurement

To capture the net radiative heat load potentially experienced by the human body in shaded spaces, *T_g_*, which accounts for the combined effects of shortwave solar radiation, longwave radiation, and air movement on the heat gain of a globe‐shaped sensor [16], was measured. Based on *T_g_*, the mean radiant temperature (*T*_mrt_) and UTCI can be calculated. In addition to the standard measurement at 1.10 m height via a measurement station, an additional black globe thermometer was installed 25 cm below the top canopy at the center of each shading canopy. This position was selected to simulate the typical distance between a person's head and the shading surface, ensuring that the globe remained consistently shaded and well ventilated throughout the experiment. *T_g_* was recorded at 1‐min intervals.

#### 2.3.3 Vertical *T_a_* Measurement

To understand the variations in vertical air temperature resulting from upward and downward energy fluxes, six Testo 174H temperature and humidity sensors were installed under each shading station in the two shaded groups. These six sensors were mounted at heights of 0.50, 1.00, 1.50, 1.75, 2.00, and 2.25 m from the ground and positioned at the center of each shading station to measure shaded air temperatures. A reference sensor was installed at the unshaded control site (without shading) at a height of 2 m to minimize ground interference and capture temperature and humidity under direct solar radiation. Each logger recorded *T_a_* and *RH* data at 1‐min intervals.

#### 2.3.4 *T_s_* and *ST* Measurement

To investigate the impact of thermal inertia and surface heating of the shading canopy, it is essential to simultaneously monitor *T_s_* and *ST*., as these surface thermal behaviors not only determine the re‐radiation capacity of the shading components but also affect the air temperature and heat stress levels within the shaded spaces. These measurements provide critical evidence for analyzing the underlying mechanisms of thermal regulation. For each shading station, temperature and humidity sensors (Testo‐175T3) connected to T‐type thermocouples were used. Thermocouples were affixed to the center of each top and side canopy to measure *T_s_*, which was recorded every 1 min. In addition, T‐type thermocouples were used to monitor *ST*., which was measured near the central underside of the shading canopy to ensure that the measured spaces remained shaded for as long as possible. In contrast, ST under sun‐exposed conditions was recorded at a control site located 10 m away from the shaded groups, ensuring that this reference site remained unshaded throughout the measurement period (Figure 3a).

### 2.4 *T*_mrt_, ∆*ST* and UTCI Calculation

The radiative heat load, quantified by *T*_mrt_, is a primary contributor to the OTC during summer daytime periods [17, 18]. A study in Nanjing, China, showed a strong correlation between UTCI and *T*_mrt_ under summer daytime conditions, with a UTCI threshold corresponding to *T*_mrt_ values exceeding 55°C for outdoor heat stress. *T*_mrt_ assessment was conducted based on Equation (1) according to ISO 7726 [19, 20].

(1) $$T_{mrt}={\left[{\left(T_{g}+273.15\right)}^{4}+\frac{1.1\times 10^{8}v^{0.6}}{\varepsilon D^{0.4}}\times \left(T_{g}-T_{a}\right)\right]}^{\frac{1}{4}}\;-273.15$$

where ε represents the emissivity of the globe (set to 0.95) and D denotes the globe diameter (set to 50 mm).

The surface cooling temperature (∆*ST*) was calculated as the temperature difference between the sunlit and shaded surfaces. Moreover, the UTCI, which integrates *T_a_*, *RH*, *V_a_*, and *T*_mrt_ was calculated. Among these four input variables, *T_a_* and *RH* were directly measured and input into the software programs, whereas *T*_mrt_ and *V_a_* were converted based on Equations (1) and (2). Measurement stations for wind speed were set at 2 m, while Equation (2) converted it to the wind speed at 10 m [21]. Moreover, to compare the thermal quality at each site, UTCI was adopted as the primary thermal stress index [20], where the final UTCI values were interpreted using the 10‐level thermal stress classification scale [21].

(2) $$v_{x}=v_{m}\;\times \frac{\log\left(\frac{x}{z_{0m}}\right)}{\log\left(\frac{m}{z_{0m}}\right)}$$

where *v_x_* represents the wind speed at a specific measurement height *x*, *m* is the actual height at which the wind speed is measured (unit: m), and z_0m_ is the surface roughness length governing momentum transfer (set to 0.01 m).

### 2.5 Data Analytical Methods

To examine the relationship between UTCI and microclimatic variables, including *T*_mrt_, *T_a_*, *V_a_*, and *T_g_*, linear regression analysis was applied   [7]. Significance was evaluated using *p* values. Statistical significance is indicated with asterisks as follows: one asterisk for *p*‐values between 0.01 and 0.05, two asterisks for p‐values between 0.001 and 0.01, and three asterisks for *p*‐values below 0.001. Cases with no significant correlation were marked as “ns” [6]. Under the same environmental conditions, this study enabled direct comparisons to assess the effectiveness of PV canopy and fabric shading in improving OTC.

In the analytical framework, apart from the variation in thermal parameters throughout the day and four periods (e.g., A, B, C, and D), particular attention was given to the time period of 10:30–13:30, corresponding to the peak hours of shading effectiveness. During this period, the central monitoring point beneath each shading station remained fully shaded, with no direct solar penetration. Therefore, this time window was considered optimal for evaluating the radiative shielding performance of the shading facilities and their influence on UTCI reduction under stable solar load conditions. Such targeted analysis allows the isolation of material‐driven effects by minimizing geometric variations in shadow projection. For broader comparative analyses, the dataset was grouped into daytime (05:30–18:35) and nighttime (18:35–05:30) periods based on the sunrise and sunset times during the measurement period. This dual‐layer temporal framework supports both dynamic and cumulative evaluations of the heat mitigation performance across different shading surface configurations.

## 3 Analysis of Heat Mitigation Performance of Artificial Shading Stations

This section analyzes the heat mitigation performance and associated mechanisms of the two types of artificial shading. Compared to the unshaded control group, the analysis revealed how the shaded stations caused differences in heat mitigation across various time periods, vertical height levels, and ground surface types.

### 3.1 Daily Variation of Microclimatic Variables

**FIGURE 4:**
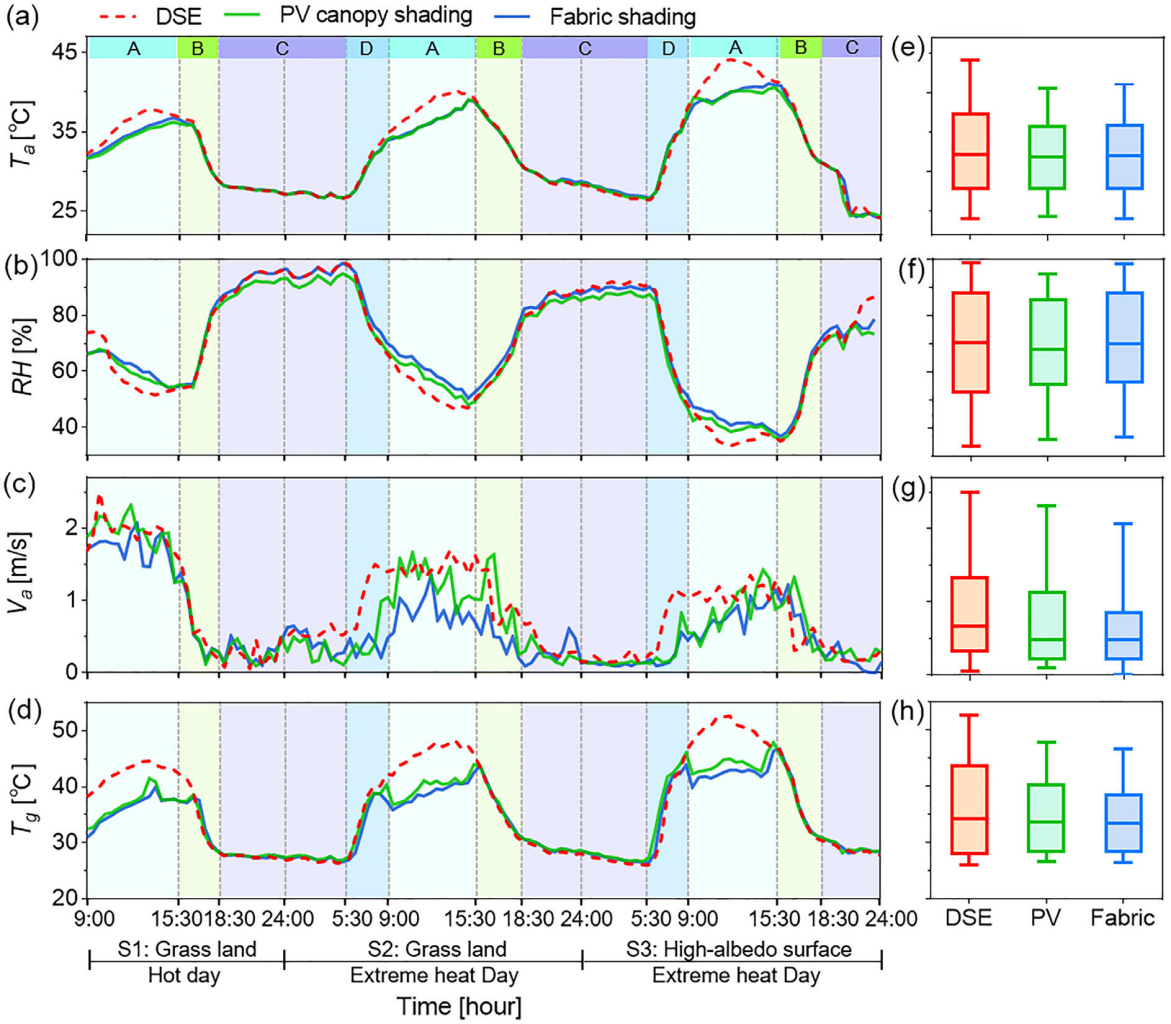
Daily variations of (a) (e) Air temperature, (b) (f) Relative humidity, (c) (g) Wind speed, and (d) (h) Black globe temperature under different spatial scenarios.

Figure 4 shows the daily variation of key microclimatic variables in the two shaded groups and one unshaded control group under the three spatial scenarios (scenario 1, 2, and 3). These variables were analyzed in the following four periods: A (midday/afternoon, 09:00–15:30), B (late afternoon, 15:30–18:35), C (night, 18:35–05:30), and D (early morning, 05:30–09:00).

#### 3.1.1 Period A‐ Midday/Afternoon

During Period A, the differences among the shaded and unshaded groups were most evident. *T_a_* in the unshaded control group was generally higher than that in the two shaded groups, especially during the extreme heat stage, with the maximum *T_a_* reduction reaching 4.8°C under fabric shading. *RH* showed an opposite pattern to *T_a_*. The unshaded group generally had the lowest *RH*, whereas fabric shading and PV canopy shading increased *RH* by up to approximately 12% and 8%, respectively. *V_a_* fluctuated during this period, and the differences among the three groups were less consistent than those of *T_a_* and *RH*. The shaded groups tended to show slightly lower *V_a_* in some scenarios, indicating a weak wind‐sheltering effect. *T_g_* responded most strongly to shading. In the unshaded group, *T_g_* exceeded 50°C during extreme heat conditions, whereas the maximum *T_g_* was approximately 48°C under PV canopy shading and no more than 47°C under fabric shading. Overall, Period A was the main stage in which shading produced clear heat mitigation, primarily by reducing radiant heat exposure and lowering *T_a_*.

#### 3.1.2 Period B‐ Late Afternoon

During Period B, solar radiation weakened, and the thermal difference among the groups gradually decreased. *T_a_* in all groups tended to decline or stabilize, and the shaded–unshaded difference became smaller than that in Period A. *RH* increased continuously with a decrease in *T_a_*. The *RH* curves of the three groups were generally close, with an overall increase of approximately 30% and a maximum inter‐group difference of only approximately 2%. *V_a_* decreased in all groups and approached a low‐speed level of approximately 0.2 m/s. The unshaded group generally maintained a relatively higher *V_a_*, followed by the PV canopy shading group, whereas fabric shading showed the lowest *V_a_*. *T_g_* decreased rapidly as solar radiation declined, and the shading‐induced *T_g_* reduction weakened accordingly. Taking scenario 2 as an example, the *T_g_* difference decreased from approximately 5°C at 15:30 to nearly 0°C around 17:00. Therefore, Period B was a transition stage: the cooling effect of shading remained observable at the beginning of the period but weakened rapidly, particularly for *T_g_*.

#### 3.1.3 Period C‐Night

During Period C, the three groups showed similar nocturnal cooling processes. *T_a_* continued to decrease, and the differences between the shaded and unshaded groups became smaller in all scenarios. *T_g_* also decreased markedly after sunset and then remained similar among the three groups, indicating that the daytime shading effect on the radiant heat load was largely reduced at night. *RH* increased steadily throughout the night. The *RH V_a_* lues under fabric shading and in the unshaded group were generally similar, whereas PV canopy shading showed slightly lower *RH* during part of this period, with a difference of approximately 4% from the other two groups. *V_a_* remained weak, mostly within approximately 0.1–0.3 m/s. The fabric shading generally showed the lowest *V_a_*, but the absolute difference was limited because the overall airflow level was low. Overall, Period C was characterized by convergence in *T_a_* and *T_g_*, increased *RH*, and weak airflow, suggesting that the heat mitigation effect of shading was no longer dominant after sunset.

#### 3.1.4 Period D‐ Early Morning

During Period D, *T_a_* began to increase again as solar radiation increased. The divergence between the shaded and unshaded groups gradually reappeared after sunrise. Over the grass‐covered surface, differences between Ta and Tg became evident at approximately 07:30. The unshaded group showed a faster increase in *T_a_*, while both shaded groups delayed the warming process. By 09:00, the maximum *T_a_* difference between shaded and unshaded groups reached approximately 2.5°C. *RH* decreased gradually with increasing *T_a_*, and fabric shading maintained slightly higher *RH* than the unshaded group, with a difference of approximately 2%. *V_a_* varied among the scenarios. In more open spaces, the unshaded group maintained a relatively higher *V_a_*, whereas fabric shading generally resulted in a lower *V_a_* due to stronger airflow obstruction. *T_g_* increased rapidly after sunrise, and the shading effect became evident. By 09:00, the maximum *T_g_* reduction was approximately 2°C under PV canopy shading and 5°C under fabric shading. On the high‐albedo surface, the divergence between *T_a_* and *T_g_* appeared approximately 0.5–1 h later than on the grass‐covered surface. Overall, Period D showed a gradual re‐establishment of the shading effect after sunrise, with a stronger response in *T_g_* than in *T_a_*.

From a whole‐day perspective, the microclimatic differences caused by shading showed a clear diurnal pattern. The strongest differences occurred during Period A, when solar radiation was intense, and shading substantially reduced *T_g_* and *T_a_* while maintaining a relatively higher *RH*. These differences weakened rapidly during Period B as solar radiation declined and nearly disappeared during Period C, when *T_a_* and *T_g_* among the three groups converged under nocturnal cooling conditions. After sunrise in Period D, the differences gradually reappeared, particularly in *T_g_*, indicating that the shading effect was closely linked to the daily variation in solar radiation. Between the two shaded groups, fabric shading produced a slightly larger maximum reduction and slightly smaller average reduction in *T_a_* and *T_g_*, generally maintained slightly higher *RH*, but it also showed a more evident airflow obstruction effect, resulting in lower *V_a_*. PV canopy shading also reduced thermal exposure, but its cooling effect was generally weaker than that of fabric shading, and its influence on airflow was relatively smaller. Therefore, the two shading stations modified the daily microclimate in different ways: fabric shading provided stronger thermal mitigation but greater wind sheltering, whereas PV canopy shading offered a more moderate cooling effect with less airflow suppression than fabric shading.

### 3.2 Daily Variation of *ST* and *T_s_*

**FIGURE 5:**
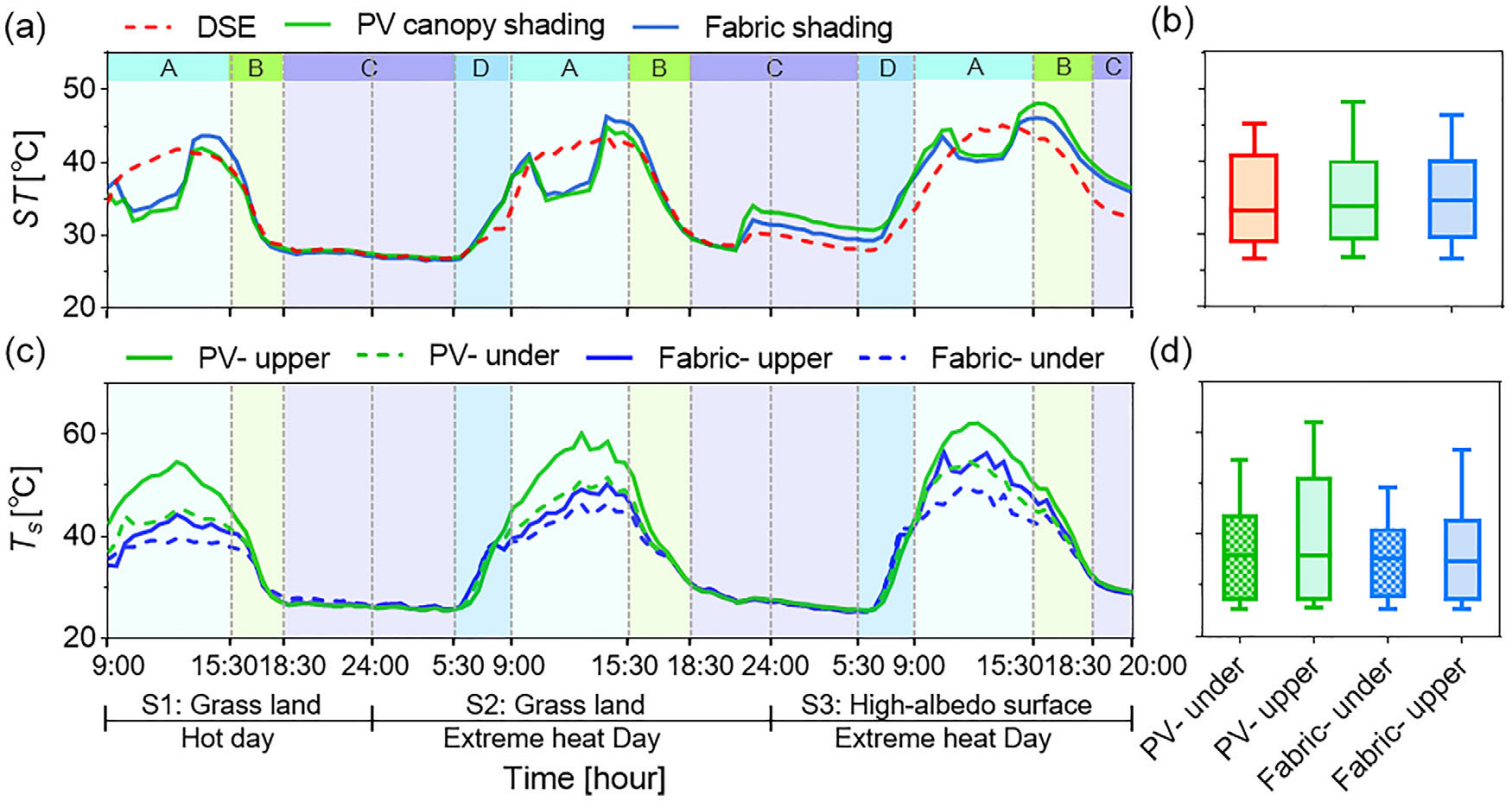
Daily variations of (a) (b) Surface temperature at the ground level and (c) (d) Surface temperature of shading canopy under different spatial scenarios.

Figure 5 shows the daily variation in the ground surface temperature (*ST*) and shading canopy surface temperature (*T_s_*) under different spatial scenarios. Panels (a) and (b) compare *ST* among the unshaded control group, PV canopy shading, and fabric shading, whereas panels (c) and (d) compare *T_s_* between the upper and underside surfaces of the PV and fabric canopies. Table 3 summarizes the mean values during the daytime (05:30–18:35) and nighttime (the remaining period of the day).

**TABLE 3:** Daytime and nighttime heat mitigation performance of the PV canopy and fabric shading in terms of ground surface temperature and canopy surface temperature.

Period	Scenario	Mean ground surface temperature (*ST*) (°C)			Mean canopy surface temperature (*T_s_*) (°C)		
		DSE	PV–DSE	Fabric–DSE	PV canopy	Fabric	PV‐Fabric
Daytime	S1	38.3	−2.8	−1.4	39.9	36.1	3.8
	S2	38.1	−1.2	−0.4	44.4	40.7	3.7
	S3	39.5	1.8	0.9	46.3	43.8	2.5
	D‐Mean	38.6	−0.7	−0.3	43.5	40.2	3.3
Night‐time	S1	28.1	−0.1	−0.4	25.7	25.9	−0.2
	S2	28.2	0.5	0.0	27.0	27.3	−0.3
	S3	30.1	2.4	2.2	27.2	27.0	0.2
	N‐ Mean	28.8	0.9	0.6	26.6	26.7	−0.1

#### 3.2.1 Period A‐ Midday/Afternoon

In Period A, *ST* increased rapidly in all scenarios. In the grassland scenarios, the shaded and unshaded groups showed broadly similar *ST* variations, although shading tended to lower *ST* during part of the daytime process. In the high‐albedo scenario, however, *ST* beneath the shaded stations remained relatively high, and PV canopy shading showed the highest *ST* near the end of Period A. This indicates that the effect of shading on *ST* was not uniform across surface types.

*T_s_* responded more clearly to solar exposure than *ST*. The upper surfaces of both canopies heated rapidly, whereas the underside surfaces remained lower. The PV upper surface consistently showed the highest *T_s_*, reaching approximately 55–60°C or higher during the hottest periods. The fabric underside generally exhibited the lowest *T_s_*. Thus, Period A was characterized by strong canopy‐surface heating, especially for the PV canopy, whereas the ground‐level *ST* response was more dependent on the scenario.

#### 3.2.2 Period B‐ Late Afternoon

During Period B, *ST* decreased as solar radiation weakened. In the grassland scenarios, the *ST* curves of the three groups gradually converged. In the high‐albedo scenario, *ST* beneath the shaded stations, especially under PV canopy shading, remained higher at the beginning of this period but then continuously declined.

*T_s_* also decreased rapidly during Period B. The upper canopy surfaces cooled from their daytime peaks, and the temperature difference between the upper and underside surfaces decreased. Although the PV canopy remained warmer than the fabric canopy during the early part of the period, this difference decreased as solar radiation declined. Overall, Period B was a rapid surface‐cooling stage for both *ST* and *T_s_*.

#### 3.2.3 Period C‐Night

During Period C, *ST* remained relatively low and stable. The *ST* curves of the three groups were close in the grassland scenarios, whereas the shaded groups were slightly warmer than the unshaded group in the high‐albedo scenario. This suggests that nighttime *ST* differences were limited on grassland but were more evident over high‐albedo surfaces.

*T_s_* also converged after sunset. The upper and underside surface temperatures of both canopies decreased to a similar low range, and the daytime differences between the PV and fabric surfaces were greatly reduced. Therefore, canopy material and surface position had much weaker effects on *T_s_* during the nighttime period.

#### 3.2.4 Period D‐Early Morning

During Period D, *ST* began to increase again after sunrise. In the grassland scenarios, the three groups were close at the beginning of the period, and differences gradually appeared with increasing solar radiation. Compared with Period A, however, the *ST* difference remained limited. In the high‐albedo scenario, the shaded groups tended to show higher *ST* after solar heating intensified.

*T_s_* increased earlier and more rapidly than *ST*, particularly on the upper surfaces of the canopy. The PV upper surface warmed fastest and became higher than the other canopy surfaces, while the fabric upper surface remained lower. The underside surfaces of both canopies increased more slowly and stayed below their corresponding upper surfaces. Thus, Period D marked the reappearance of vertical surface temperature differences within the shading structures.

Overall, the surface‐temperature results showed distinct day‐night and scenario‐dependent patterns. During daytime, the mean *ST* of the unshaded group was 38.6°C. PV canopy shading and fabric shading reduced the daytime mean *ST* by only 0.7°C and 0.3°C on average, respectively. This limited overall reduction resulted from contrasting responses among scenarios: in S1 and S2, both shading facilities reduced *ST*, with the PV canopy showing reductions of 2.8°C and 1.2°C and fabric shading showing reductions of 1.4°C and 0.4°C; in S3, however, *ST* increased by 1.8°C under PV canopy shading and 0.9°C under fabric shading. At night, the shaded groups were slightly warmer on average, with *ST* increases of 0.9°C under PV canopy shading and 0.6°C under fabric shading, mainly because of the larger nighttime increase in S3. For *T_s_*, the PV canopy was clearly warmer than the fabric canopy during daytime, with a mean difference of 3.3°C. At night, this difference almost disappeared, with nighttime mean *T_s_* values of 26.6°C for the PV canopy and 26.7°C for the fabric canopy. Consequently, the PV canopy generated stronger daytime canopy‐surface heating, whereas fabric shading helped maintain lower canopy surface temperatures. For ground‐level *ST*, the mitigation effect of both shading facilities was limited and strongly dependent on the underlying surface type.

### 3.3 Vertical Temperature Profiles Under Shading Stations

**FIGURE 6:**
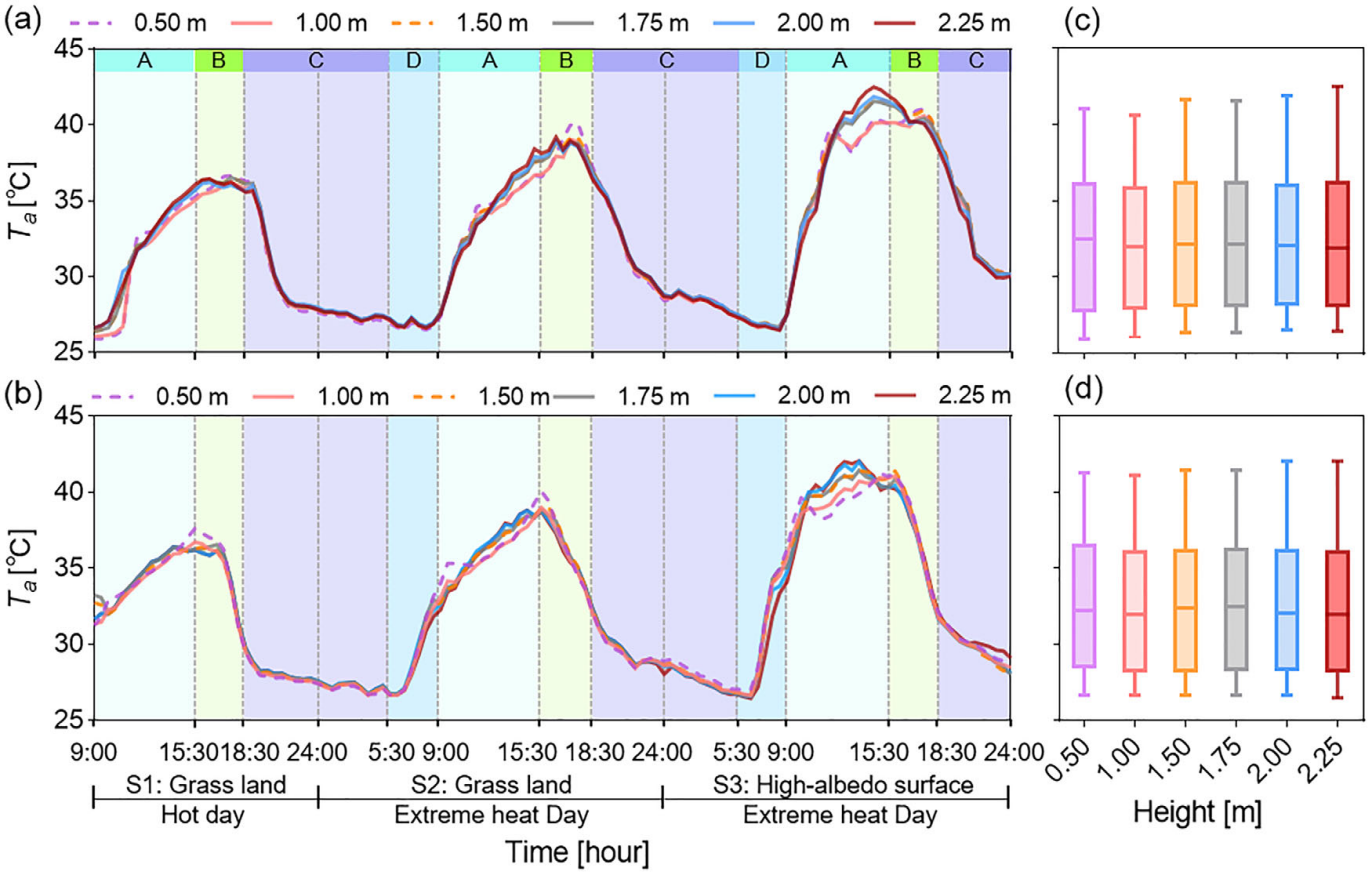
Daily variations of vertical air temperature under (a) (c) PV canopy shading, (b) (d) Fabric shading.

Figure 6 shows the vertical air temperature profiles under PV canopy and fabric shading at six measurement heights: 0.50, 1.00, 1.50, 1.75, 2.00, and 2.25 m. Panels (a) and (c) correspond to PV canopy shading, while panels (b) and (d) correspond to fabric shading. Table 4 summarizes the mean vertical air temperature during the daytime (05:30–18:35) and nighttime (the remaining period of the day). Since Figure 6 does not include the unshaded control group, this section focuses on the vertical temperature differences inside the two shading stations rather than the direct temperature reduction relative to the exposed environment.

**TABLE 4:** Daytime and nighttime performance of the PV canopy and fabric shading in terms of vertical air temperature.

Period	Scenario	Vertical air temperature under PV (°C)						Vertical air temperature under Fabric (°C)					
		0.50 m	1.00 m	1.50 m	1.75 m	2.00 m	2.25 m	0.50 m	1.00 m	1.50 m	1.75 m	2.00 m	2.25 m
Day‐time	S1	32.6	32.4	33.0	33.0	33.0	33.0	33.0	32.7	33.1	33.5	33.2	33.2
	S2	35.1	34.8	35.2	35.1	35.1	35.1	35.2	34.8	35.1	35.1	35.2	35.0
	S3	37.5	37.2	37.9	37.7	37.7	37.8	37.5	37.4	37.8	37.6	37.7	35.0
	D‐Mean	35.1	34.8	35.4	35.3	35.3	35.3	35.2	35.0	35.3	35.4	35.4	34.4
Night‐time	S1	26.7	26.8	26.9	26.9	27.0	27.0	26.9	26.8	26.9	27.0	27.0	27.0
	S2	28.1	28.2	28.3	28.4	28.4	28.3	28.3	28.3	28.4	28.4	28.5	28.4
	S3	28.5	28.3	28.5	28.5	28.4	28.3	28.8	28.6	28.6	28.5	28.5	28.3
	N‐ Mean	27.8	27.8	27.9	27.9	27.9	27.9	28.0	27.9	28.0	28.0	28.0	27.9

Under PV canopy shading, the vertical temperature differences were generally small, but a weak height‐related pattern was observed during the daytime. In S1, the daytime mean *T_a_* ranged from 32.4°C at 1.00 m to 33.0°C at 1.50–2.25 m, with a maximum vertical difference of 0.6°C. In S2, the corresponding values ranged from 34.8°C to 35.2°C, with a maximum difference of 0.4°C. In S3, the vertical difference was slightly larger, ranging from 37.2°C at 1.00 m to 37.9°C at 1.50 m. These results indicate that PV canopy shading produced only weak vertical stratification, with the middle and upper layers being slightly warmer than the lower layer during the daytime.

Under fabric shading, the vertical distribution of *T_a_* was generally uniform, although its height‐dependent pattern differed from that under PV canopy shading. In S1, daytime mean *T_a_* ranged from 32.7°C at 1.00 m to 33.5°C at 1.75 m, with a maximum vertical difference of 0.8°C. In S2, the vertical difference was very small, with mean *T_a_* varying only between 34.8°C and 35.2°C. In S3, most measurement heights remained within a narrow range of 37.4–37.8°C, except for the 2.25 m height, where the mean value was lower. Therefore, fabric shading did not exhibit a consistent upper‐layer warming pattern. Its vertical temperature field was generally uniform, with only local height‐dependent deviations in the high‐albedo surface scenario.

A comparison between the two shading stations showed that both facilities produced limited vertical *T_a_* differences within the occupied space. During daytime, the mean *T_a_* across all scenarios ranged from 34.8°C to 35.4°C under PV canopy shading and from 34.4°C to 35.4°C under fabric shading. The PV canopy showed a slightly more evident warming tendency in the middle‐to‐upper layers, especially around 1.50–2.25 m, whereas fabric shading showed a less systematic vertical pattern. This suggests that the two shading materials affected the vertical air temperature field in different ways: PV canopy shading tended to form weak upper‐layer warming, whereas fabric shading maintained a more mixed vertical temperature distribution.

At night, the vertical temperature differences became much weaker under both shading stations. Under PV canopy shading, nighttime mean *T_a_* varied within 26.7–27.0°C in S1, 28.1–28.4°C in S2, and 28.3–28.5°C in S3. Under fabric shading, the corresponding ranges were 26.8–27.0°C, 28.3‐28.5°C, and 28.3–28.8°C. The maximum vertical difference was mostly within 0.5 °C, indicating that the height‐related temperature differences almost disappeared after sunset. Therefore, the vertical thermal influence of both shading stations was mainly reflected during the daytime, whereas the nighttime air temperature was nearly vertically uniform.

From the perspective of different scenarios, the overall temperature changed more clearly than the vertical gradient. S1 had the lowest *T_a_*, S2 had a higher Ta, and S3 had the highest *T_a_* under both shading stations. However, within each scenario, the differences among the six heights were limited. This indicates that the surface type and background thermal conditions mainly determined the overall air temperature level, whereas the shading structures only caused weak vertical redistribution of *T_a_* within their lower spaces. Overall, the vertical temperature profiles under both shading stations were characterized by small, height‐dependent differences. PV canopy shading showed slightly stronger middle‐to‐upper layer warming, whereas fabric shading maintained a relatively uniform vertical temperature distribution.

**FIGURE 7:**
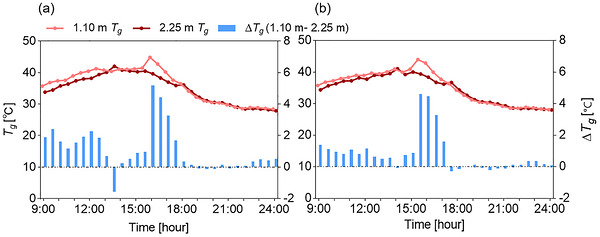
The black globe temperature variations at 1.10m and 2.25m height below the (a) PV canopy shading and (b) Fabric shading at 09:00–24:00 on 2024.08.23.

In addition to air temperature, the black globe temperature was measured at 1.10 m and 2.25 m to further examine the vertical difference in radiant thermal conditions under the shading stations. As shown in Figure 7, *T_g_* at 1.10 m was generally higher than that at 2.25 m during the daytime under both PV canopy and fabric shading. This pattern differs from the vertical air temperature results, where the height‐related differences were generally weak, indicating that the vertical variation in radiant heat exposure was more evident than that in air temperature.

Under PV canopy shading, the difference between 1.10 m and 2.25 m was limited in the morning but increased in the afternoon. The maximum vertical *T_g_* difference reached approximately 4–5°C, after which the two curves gradually converged in the evening. Under fabric shading, a similar pattern was observed, but the maximum difference was slightly smaller, generally around 3–4°C. Therefore, both shading stations showed daytime vertical differences in the black globe temperature, with the PV canopy shading producing a slightly stronger vertical *T_g_* gradient than the fabric shading.

Overall, Figure 7 indicates that the radiant thermal environment varied more clearly with height than with air temperature. Although *T_a_* remained relatively uniform within the shaded spaces, *T_g_* at pedestrian height was evidently higher than that near the upper layer during the daytime. This suggests that the two shading stations affected not only the air temperature field but also the vertical distribution of radiant heat. Compared with PV canopy shading, fabric shading maintained a weaker vertical *T_g_* difference.

In summary, the vertical thermal environment is not a simple cooling space but rather a stratified system with competing thermal forces. While the ambient air at pedestrian height remains cooler, the human body is simultaneously subjected to a compounded radiative burden: lingering upward longwave radiation from the underlying surface and amplified downward longwave emissions from the solar‐heated canopy overhead. This has profound implications for urban design; to optimize outdoor thermal comfort, a holistic and systems‐based approach is necessary. Shading design must be integrated with ground surface treatment (e.g., using vegetation or low‐emissivity materials) to mitigate the elevated radiant load at the occupant level. Simply installing shading facilities over conventional heat‐absorbing pavements represents an incomplete and potentially suboptimal solution for achieving thermal comfort.

### 3.4 Microclimatic Analysis During Full‐Shading Period (10:30–13:30)

**FIGURE 8:**
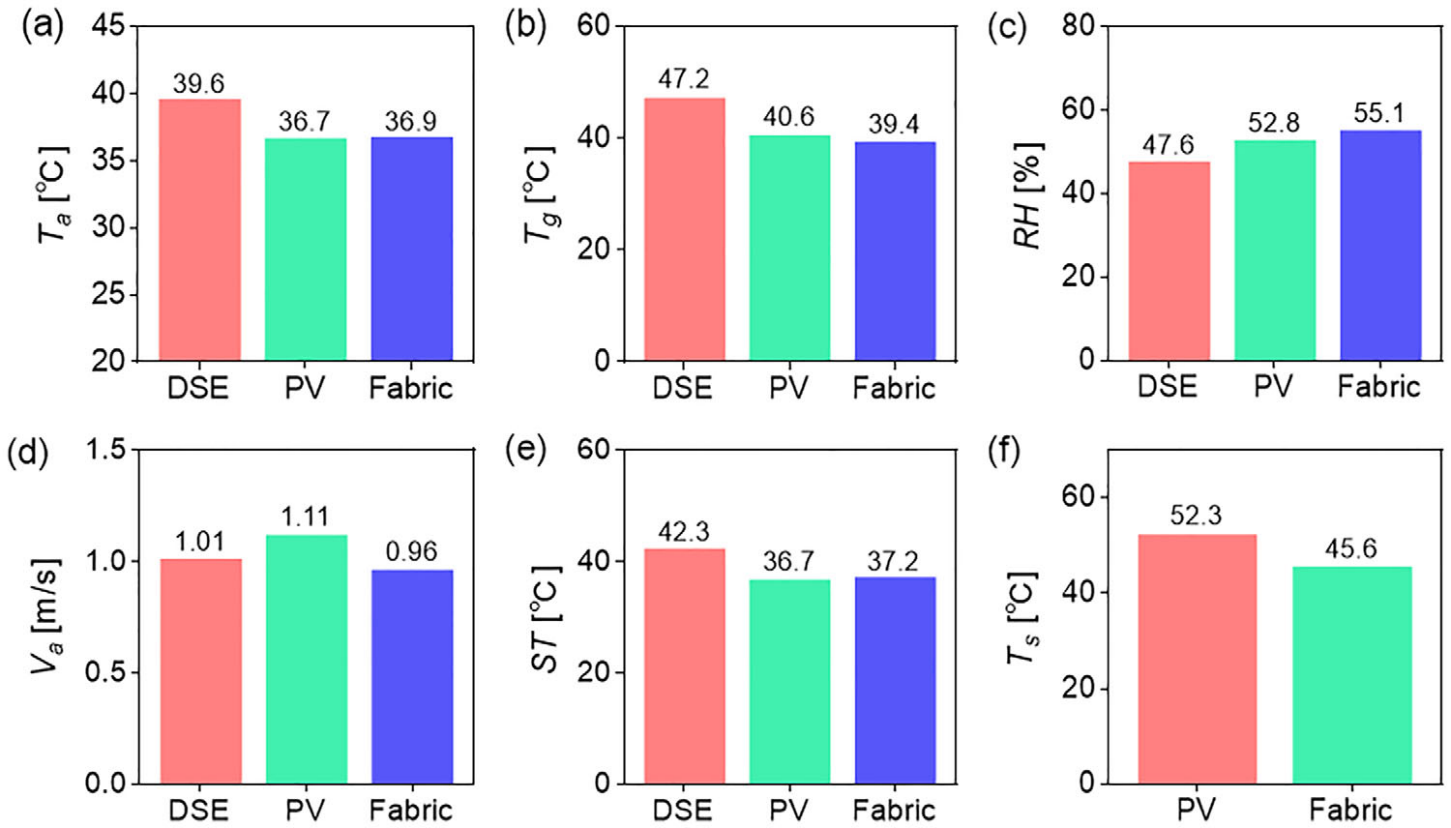
Mean values of microclimatic variables at 10:30–13:30 during the observation period. (a) 1.10‐m air temperature, (b) Black globe temperature, (c) Relative humidity, (d) Wind speed, (e) Surface temperature at the ground level, (f) Surface temperature of shading canopy.

During the full‐shading period from 10:30 to 13:30, the two shaded stations produced clear differences in the local microclimate compared with the unshaded control group. As shown in Figure 8a, the mean *T_a_* at 1.10 m was 39.6 °C under unshaded conditions, whereas it decreased to 36.7°C under PV canopy shading and 36.9°C under fabric shading. This corresponded to reductions of 2.9°C and 2.7°C, respectively. The reduction in *T_g_* was more pronounced. Figure 8b shows that *T_g_* decreased from 47.2°C in the unshaded group to 40.6°C under PV canopy shading and 39.4°C under fabric shading, with reductions of 6.6°C and 7.8°C, respectively. This indicates that the heat mitigation effect during the full‐shading period was more strongly reflected in the radiant thermal environment than in the air temperature.

*RH* also increased under shaded conditions. As shown in Figure 8c, *RH* increased from 47.6% in the unshaded group to 52.8% under PV canopy shading, and 55.1% under fabric shading. In contrast, the effect on *V_a_* differed between the two shaded stations. Figure 8d shows that *V_a_* increased from 1.01 m/s in the unshaded group to 1.11 m/s under PV canopy shading, whereas it decreased slightly to 0.96 m/s under fabric shading. This suggests that the PV canopy did not suppress airflow during the full‐shading period, whereas the fabric‐shaded station showed a weak wind‐sheltering effect.

At the surface level, both shading stations reduced *ST*. As shown in Figure 8e, *ST* decreased from 42.3°C in the unshaded group to 36.7°C under PV canopy shading and 37.2°C under fabric shading. However, Ts differed clearly between the two shading materials. Figure 8f shows that the mean Ts of the PV canopy was 52.3°C, which was 6.7°C higher than that of the fabric canopy. Therefore, although both shading stations reduced *T_a_*, *T_g_*, and *ST*, the PV canopy accumulated more surface heat than the fabric canopy during the full‐shading period.

**FIGURE 9:**
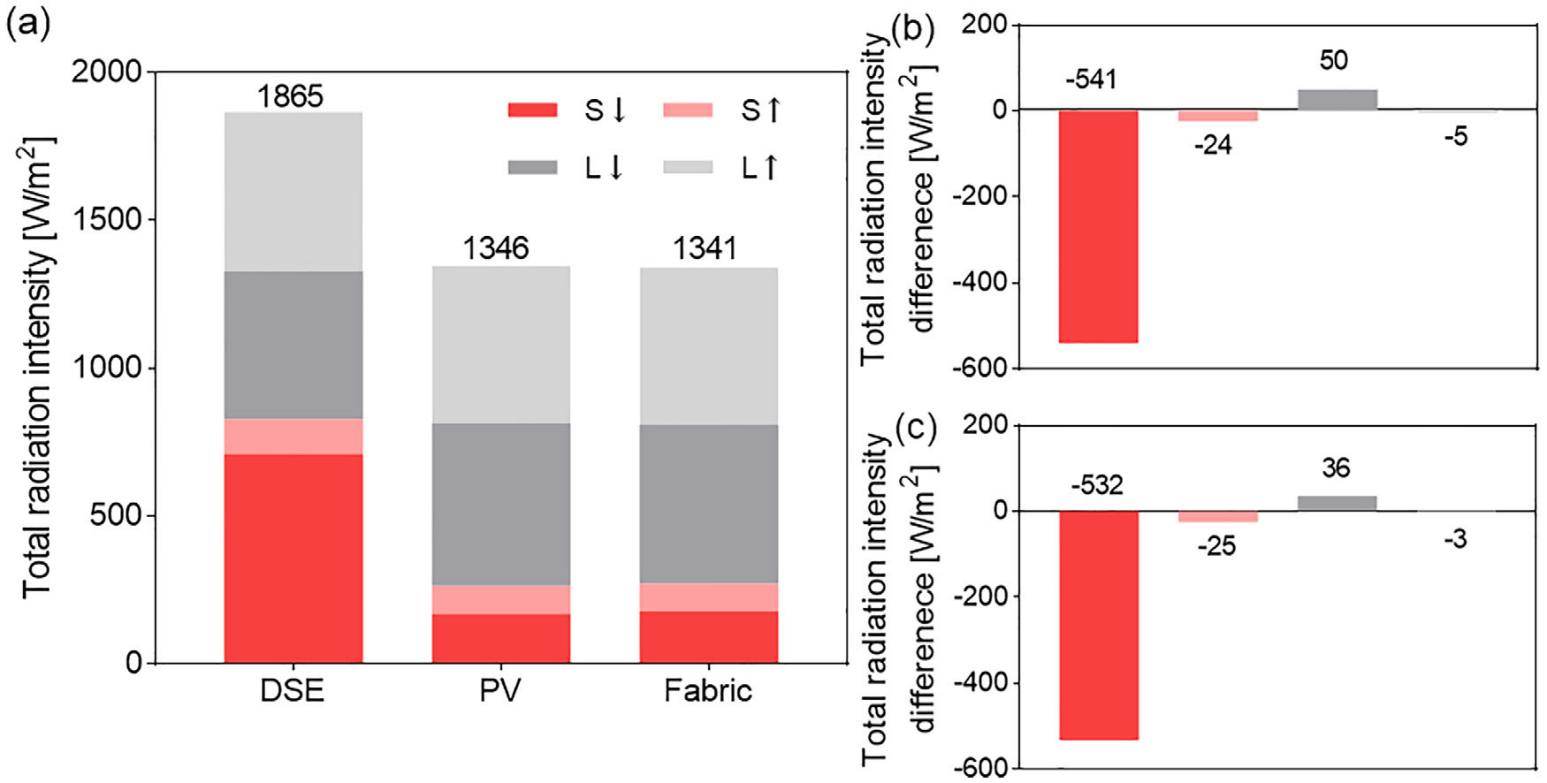
Mean (a) downward shortwave (red), upward shortwave (yellow), downward longwave (grey), and upward longwave (light grey) radiation. These are the average data observed from 10:30‐13:30 during the observation period. Differences in the mean radiation (b) between under the PV shading and in direct sunlight, and (c) between under the fabric shading and in direct sunlight.

**FIGURE 10:**
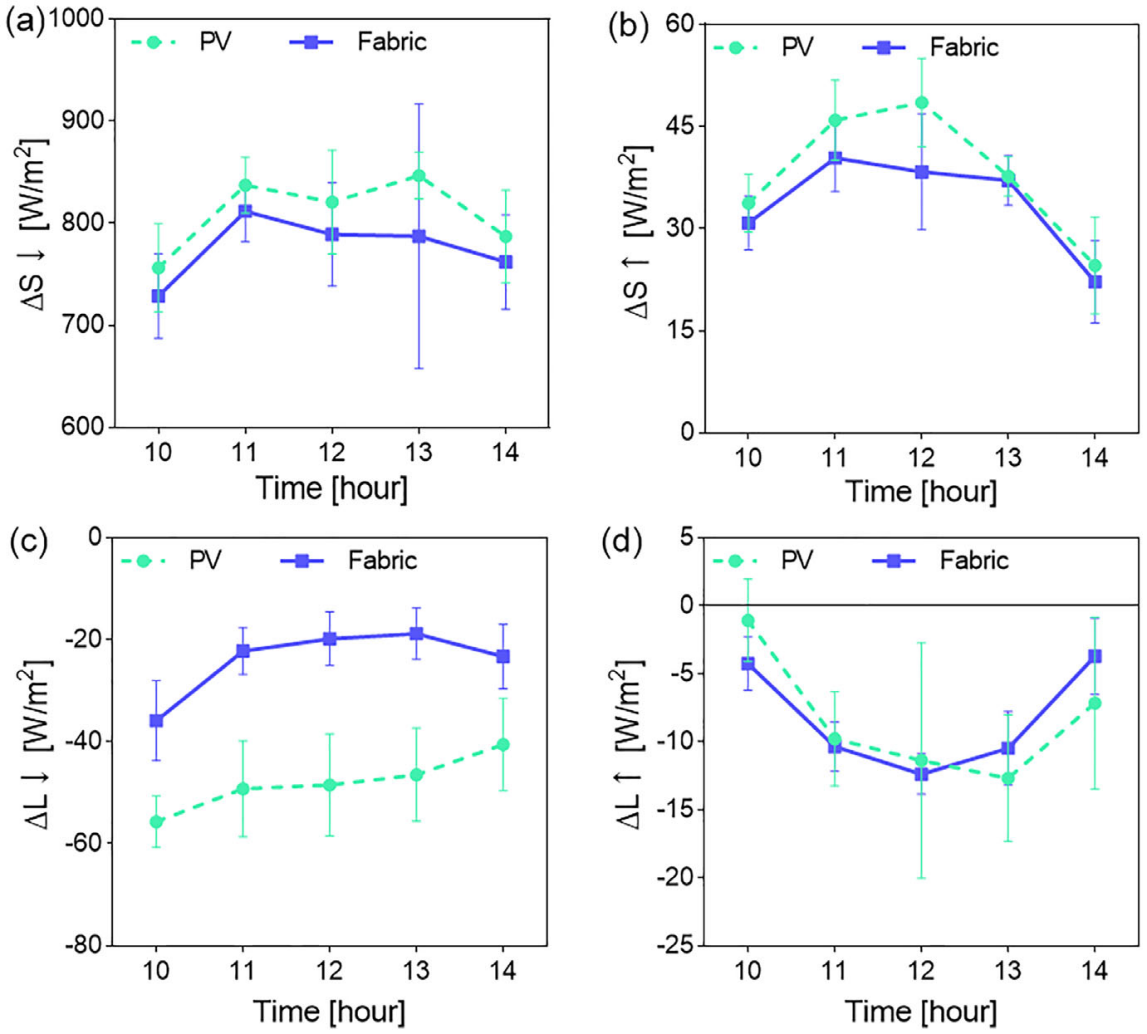
Differences in the mean radiation per hour at 10:30–13:30. (a) Downward shortwave, (b) Upward shortwave, (c) Downward longwave, and (d) Upward longwave radiation.

These differences can be explained by the changes in the radiative components shown in Figures 9 and 10. Figure 9a shows that the total radiation intensity decreased from 1865 W/m^2^ in the unshaded group to 1346 W/m^2^ under PV canopy shading and 1341 W/m^2^ under fabric shading, respectively. Thus, both the shading stations reduced the total radiative load by more than 500 W/m^2^, and the difference between the two shaded groups was very small. The dominant contributor was the reduction in downward shortwave radiation. Compared with direct sunlight, downward shortwave radiation decreased by 541 W/m^2^ under PV canopy shading and 532 W/m^2^ under fabric shading. This large reduction explains the marked decrease in *T_g_* and *ST* under both shaded conditions.

However, the two shading stations differed in their longwave response. Downward longwave radiation increased by 50 W/m^2^ under PV canopy shading and by 36 W/m^2^ under fabric shading, whereas the changes in upward longwave radiation were small. This indicates that part of the shortwave radiation reduction was offset by additional longwave radiation emitted from the heated canopy surfaces, especially under the PV canopy. This is consistent with the higher *T_s_* of the PV canopy shown in Figure 8f. Therefore, although the PV canopy slightly reduced *ST* and maintained higher *V_a_*, its hotter canopy surface enhanced downward longwave radiation, which partly weakened the radiant cooling effect. In contrast, fabric shading resulted in a lower canopy surface temperature and a smaller increase in longwave radiation, which helps explain the lower *T_g_* during the full‐shading period.

Figure 10 further shows the hourly variation in the radiative differences during the observation period. The differences in downward shortwave radiation remained much larger than those in longwave radiation throughout 10:30–13:30, confirming that shortwave radiation interception was the primary mechanism of heat mitigation for both shading stations. PV canopy shading generally showed a slightly larger shortwave radiation difference than fabric shading, whereas the longwave‐related difference was more evident under the PV canopy. This combined effect explains why the two shading stations had similar reductions in total radiation but produced different microclimatic responses: fabric shading achieved a slightly greater reduction in *T_g_* and higher *RH*, whereas PV canopy shading maintained slightly lower *ST* and higher *V_a_* but had a warmer canopy surface.

### 3.5 Comparison of Diurnal and Nocturnal Heat Mitigation Performance Between PV Canopy and Fabric Shading

To assess the overall microclimatic regulation capacity of artificial shading stations, this subsection compares the heat mitigation performance of the PV canopy and fabric shading during both daytime (from sunrise to sunset, 05:30–18:35) and nighttime (from sunset to sunrise,18:35–05:30). The three assessment indicators, *T_a_* and *T_g_*, are presented in Table 5.

**TABLE 5:** Daytime and nighttime maximum and minimum heat mitigation performance of PV canopy and fabric shading in *T_a_*, and *T_g_*.

Time	Scenario	Mean T_a_ (°C)			Mean *T*_g_ (°C)		
		DSE	PV‐ DSE	Fabric‐ DSE	DSE	PV‐ DSE	Fabric‐ DSE
Day‐time	S1	34.5	−0.3	−1.0	41.0	−4.4	−4.7
	S2	36.1	−1.2	−1.3	42.0	−3.4	−4.2
	S3	38.7	−1.5	−1.3	44.5	−2.7	−3.9
	D‐Mean	36.4	−1.0	−1.2	42.5	−3.5	−4.3
Night‐time	S1	28.0	0.0	0.1	27.7	27.8	27.7
	S2	28.2	0.0	0.1	27.9	28.4	28.3
	S3	27.1	0.0	0.3	27.6	28.4	28.2
	N‐Mean	27.8	0.0	0.2	27.7	28.2	28.1

*Note*: the maximum value(s) in each column is underlined and the minimum value(s) bold.

During daytime, both shading stations reduced *T_a_*, but the magnitude of air‐temperature reduction was relatively limited. The daytime mean *T_a_* of the unshaded group was 36.4°C. Compared with the unshaded condition, PV canopy shading reduced *T_a_* by 1.0°C on average, whereas fabric shading reduced *T_a_* by 1.2°C. Across the three scenarios, the *T_a_* reduction under PV canopy shading increased from 0.3°C in S1 to 1.2°C in S2 and 1.5°C in S3. Under fabric shading, the corresponding reductions were 1.0°C, 1.3°C, and 1.3°C. This indicates that both shading stations provided a modest daytime cooling effect on *T_a_*, with fabric shading showing a slightly larger average reduction, while PV canopy shading showed the strongest *T_a_* reduction in S3.

The daytime reduction in *T_g_* was more pronounced than that in *T_a_*. The daytime mean *T_g_* of the unshaded group was 42.5°C. PV canopy shading reduced *T_g_* by 3.5°C on average, whereas fabric shading reduced *T_g_* by 4.3°C. In S1, *T_g_* decreased by 4.4°C under PV canopy shading and 4.7°C under fabric shading. In S2, the reductions were 3.4 °C and 4.2 °C, respectively. In S3, the reductions were 2.7°C and 3.9°C. These results show that the shading effect was more evident in *T_g_* than in *T_a_*, confirming that the main daytime cooling contribution of the shaded stations was related to the mitigation of radiant heat exposure. Fabric shading consistently produced a larger *T_g_* reduction than PV canopy shading in all three scenarios.

During nighttime, the influence of the shading stations on *T_a_* was very weak. The nighttime mean *T_a_* of the unshaded group was 27.8°C. PV canopy shading showed almost no nighttime effect on *T_a_*, with an average difference of 0.0°C across the three scenarios. fabric shading showed a slight warming effect, with an average increase of 0.2°C. Specifically, fabric shading increased nighttime *T_a_* by 0.1°C in S1 and S2, and by 0.3°C in S3. Therefore, the nighttime air temperature effect of both shading stations was much weaker than their daytime effect, and fabric shading showed only a minor heat‐retention tendency.

For nighttime *T_g_*, the differences among the three groups were also small, but the shaded groups were generally slightly higher than those of the unshaded group. In S1, *T_g_* was almost identical among the three groups, with values around 27.7–27.8°C. In S2, *T_g_* increased from 27.9°C in the unshaded group to 28.4°C under PV canopy shading and 28.3°C under fabric shading. In S3, *T_g_* increased from 27.6°C to 28.4°C and 28.2°C under PV canopy shading and fabric shading, respectively. On average, nighttime *T_g_* was 27.7°C in the unshaded group, 28.2°C under PV canopy shading, and 28.1°C under fabric shading. This suggests that the shading structures had a slight nighttime warming effect on the radiant thermal environment, particularly under the PV canopy shading. Overall, the comparison between daytime and nighttime shows that the cooling effect of both shading stations was mainly concentrated during daytime. For *T_a_*, the daytime reductions were modest, with average decreases of 1.0°C under PV canopy shading and 1.2°C under fabric shading. For *T_g_*, the reductions were much larger, reaching 3.5°C and 4.3°C, respectively. At night, both *T_a_* and *T_g_* differences became very small. PV canopy shading was nearly neutral for *T_a_* but slightly increased *T_g_*, whereas fabric shading produced a minor warming effect on both *T_a_* and *T_g_*. Between the two shading stations, fabric shading provided slightly stronger daytime heat mitigation, particularly for *T_g_*, whereas PV canopy shading had a somewhat stronger effect on maintaining *T_g_* at night.

## 4 Analysis of Outdoor Thermal Comfort Under DSE, PV and Fabric Shading Conditions

### 4.1 Comparison of *T*_mrt_ Under Shaded and Unshaded Conditions

**FIGURE 11:**
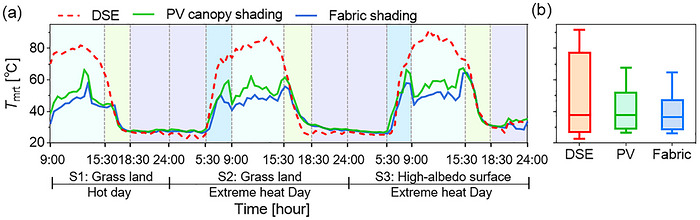
Daily variations of mean radiant temperature under different spatial scenarios.

Figure 11 shows the daily variation in *T*_mrt_ under unshaded conditions, PV canopy shading, and fabric shading across the three spatial scenarios. Compared with the microclimatic variables discussed above, *T*_mrt_ showed a much stronger response to direct solar exposure and shading. The difference between shaded and unshaded conditions was mainly concentrated during the daytime, while the three groups became close after sunset.

#### 4.1.1 Period A‐Midday/Afternoon

During Period A, *T*_mrt_ under unshaded conditions increased rapidly and remained at a high level in all scenarios. In S1, the unshaded *T*_mrt_ generally stayed around 70–80°C during the daytime heating stage. In S2 and S3, the unshaded group reached even higher levels, with peak values approaching or exceeding 80°C. In contrast, both shaded groups maintained much lower *T*_mrt_ throughout most of Period A. Under PV canopy shading, *T*_mrt_ was generally around 45–65°C, while under fabric shading, it was mostly lower, approximately within 40–55°C. Therefore, both shading stations substantially reduced daytime radiant heat exposure, and fabric shading generally showed a stronger reduction than PV canopy.

#### 4.1.2 Period B‐Late afternoon

During Period B, *T*_mrt_ decreased rapidly in all groups as solar radiation weakened. The unshaded group still remained higher than the shaded groups at the beginning of this period, but the difference narrowed quickly. Under PV canopy and fabric shading, *T*_mrt_ also decreased, and the two shaded curves gradually approached each other. By the end of Period B, the *T*_mrt_ values of the three groups were similar, indicating that the shading‐induced difference in radiant heat exposure weakened rapidly after direct solar radiation declined.

#### 4.1.3 Period C‐Night

During Period C, *T*_mrt_ remained low and stable. The curves of the unshaded group, PV canopy shading, and fabric shading almost overlapped in most nighttime intervals, generally staying around 25–30°C. This indicates that after sunset, the difference in radiant thermal conditions among the three groups became very small. Therefore, the heat mitigation effect of shading on *T*_mrt_ was mainly a daytime phenomenon.

#### 4.1.4 Period D‐Early Morning

During Period D, *T*_mrt_ began to increase again after sunrise. The unshaded group increased more rapidly than the shaded groups, particularly when direct solar exposure became stronger. Under both shading stations, the morning increase in *T*_mrt_ was delayed and suppressed. The difference between shaded and unshaded conditions gradually reappeared, but it was still smaller than that observed during the midday/afternoon period.

The boxplot in Figure 11b further confirms the overall differences among the three groups. The unshaded condition had the widest *T*_mrt_ distribution and the highest upper range, reflecting a strong daytime exposure to solar radiation. Both shading stations reduced the overall *T*_mrt_ and narrowed the distribution range. Between the two shaded groups, fabric shading generally showed a slightly lower *T*_mrt_ distribution than PV canopy shading, indicating a stronger ability to reduce radiant heat exposure for the former. Overall, the results show that the main contribution of shading to thermal mitigation was the reduction of the daytime radiant heat load, with fabric shading performing slightly better than PV canopy shading in terms of *T*_mrt_ reduction.

### 4.2 Comparison of UTCI Under DSE, PV Canopy, and Fabric Groups

**FIGURE 12:**
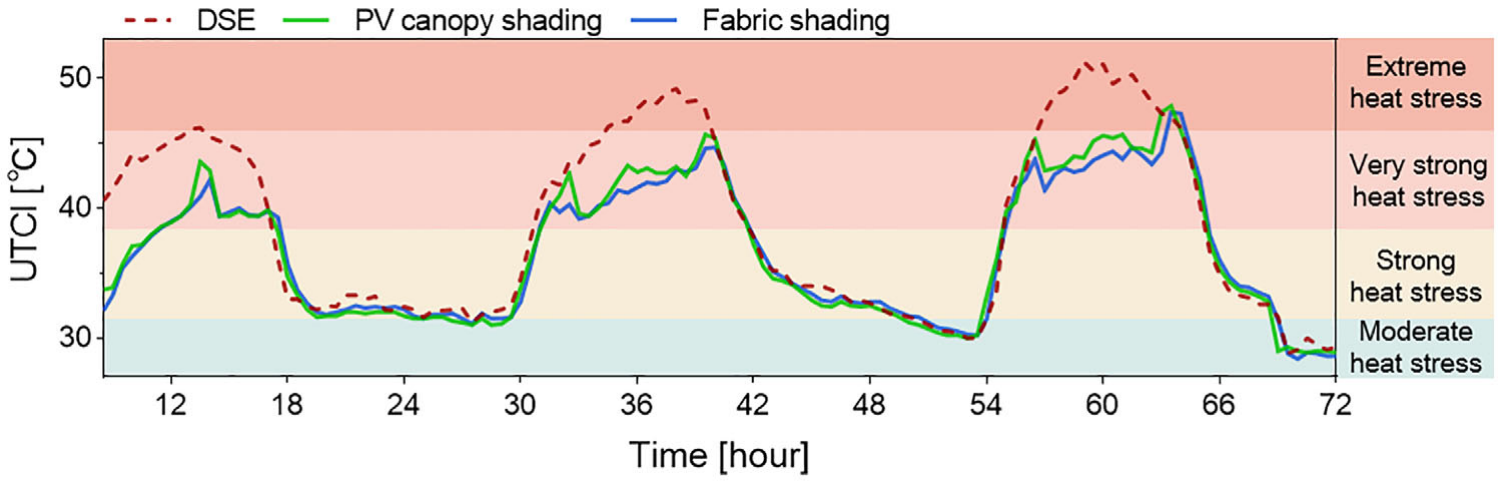
Comparison of UTCI levels in three consecutive summer days.

**FIGURE 13:**
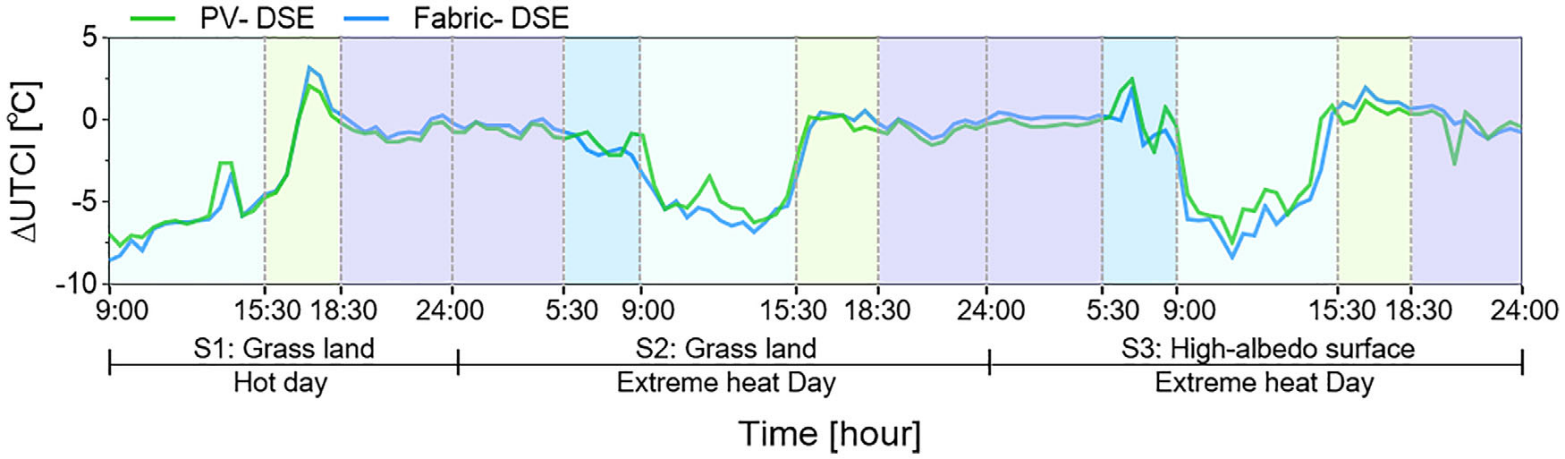
UTCI difference between two shading scenarios and DSE scenario.

Figure 12 shows the UTCI variations in the unshaded control group (DSE), PV canopy shading group, and fabric shading group during the three consecutive summer days. Figure 13 further presents the UTCI differences between the shaded groups and the DSE group, where negative values indicate a UTCI reduction under shading. The UTCI levels were interpreted according to the heat stress categories shown in Figure 12.

In S1, corresponding to the grassland scenario on a hot day, the UTCI in the DSE group increased markedly during the daytime and reached a very strong heat stress level. Both PV canopy and fabric shading reduced the UTCI during most of the daytime period. As shown in Figure 13, the reduction was generally about 5–8°C before the late afternoon, with fabric shading slightly stronger than PV canopy shading for much of this period. After sunset, the three groups became close, indicating that the shading effect on the UTCI was mainly limited to the daytime.

In S2, corresponding to the grassland scenario on an extreme heat day, the DSE group experienced extreme heat stress during the hottest period. Both shading stations clearly reduced the UTCI, keeping the shaded groups mostly below the DSE curve. The UTCI reduction was generally about 4–6°C during the main daytime heating period, and fabric shading again showed a slightly larger reduction at several moments. In the evening and nighttime, the UTCI difference among the three groups decreased.

In S3, corresponding to the high‐albedo surface scenario on an extreme heat day, the DSE group again reached the extreme heat stress range during the daytime. The shaded groups also experienced high UTCI, but their values remained lower than the DSE group during the main daytime period. The reduction was approximately 5–8°C from morning to early afternoon, with fabric shading generally producing slightly lower UTCI than PV canopy shading. During transition periods and nighttime, the differences among the three groups weakened.

Overall, the unshaded group exhibited peak UTCI values exceeding 46°C, reaching the “extreme heat stress” category during the hottest hours of the day. In comparison, both fabric and PV canopy shading stations substantially reduced UTCI. In Scenario 3, the fabric shading achieved a greater cooling effect‐ up to 7.5°C lower during peak hours, whereas PV shading reduced UTCI by up to 6.1°C. Although shading did not fully prevent exposure to “very strong heat stress,” it effectively attenuated the intensity and duration of thermal stress. These results underscore the importance of solar control facilities in improving outdoor thermal comfort during hot periods.

### 4.3 UTCI Reduction Performance During Full‐Shading Period (10:30–13:30)

**FIGURE 14:**
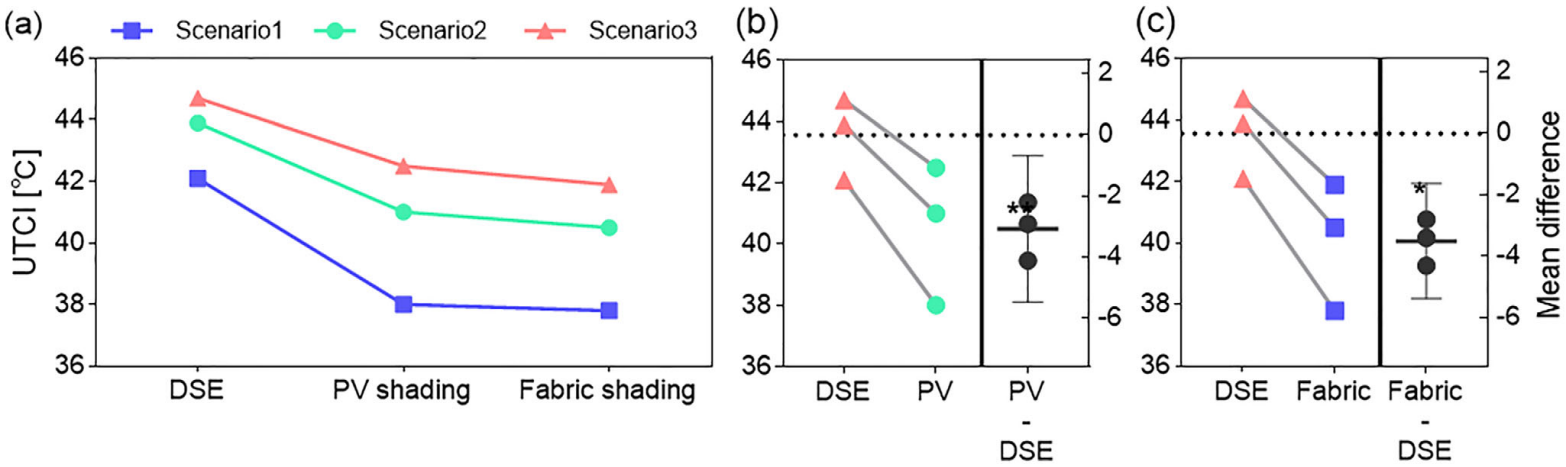
Average UTCI at 10:30–13:30 during the midday period. (a) Mean value, (b) Mean difference between DSE and PV canopy shading, and (c) Mean difference between DSE and fabric shading.

A statistical comparison of the UTCI under different shading stations during the full‐shading period was conducted. Figure 14 displays the average UTCI values and standard deviations observed in the unshaded, PV canopy shading, and fabric shading groups. In the unshaded group, the average UTCI during the midday period reached 47.5°C, which fell within the category of “extreme heat stress” (UTCI > 46°C). In comparison, the UTCI under both fabric and PV canopy shading averaged 41.2°C and 42.0°C, respectively, corresponding to reductions of 6.3°C and 5.5°C relative to unshaded spaces. Welch's t‐test confirmed that the shaded groups had significantly lower UTCI values than the unshaded group (*p* < 0.001). Posthoc comparisons using the Bonferroni correction further verified the statistical significance of these differences. Overall, both artificial shading facilities significantly alleviated thermal stress during midday on hot days, with fabric shading demonstrating slightly better performance than PV canopy shading.

In S1, fabric shading demonstrated a slightly greater cooling effect, reducing the average UTCI by 4.5°C compared to unshaded group, whereas PV canopy shading resulted in a UTCI reduction of 4.3°C. Under more intense heat conditions (S2 and S3), fabric shading outperformed PV canopy shading, with an average advantage of approximately 0.5°C to 1.5°C, indicating better effectiveness in mitigating thermal stress. The average UTCI reduction and error ranges under each condition are shown in Figure 14. The results suggest that while both shading stations achieved comparable average UTCI reductions, Fabric shading generally achieved more consistent UTCI reductions across different spatial scenarios, indicating greater performance stability under highly exposed conditions. Overall, the full‐shading period was a critical time window for assessing the cooling capacities of the different shading stations. The performance difference between the two shading stations was relatively small under the typical hot day scenario, whereas fabric shading provided greater thermal stress mitigation under extreme heat conditions.

### 4.4 Comparison of Cooling Performance Between PV Canopy and Fabric Shading

To assess the overall thermal comfort regulation capacity of the two shading stations, this subsection compares the heat mitigation performance of PV canopy shading and fabric shading during both daytime and nighttime in terms of *T*_mrt_ and UTCI. Daytime was defined as 05:30–18:35, and nighttime was defined as 18:35–05:30. The mean values and shading‐induced differences are presented in Table 6.

**TABLE 6:** Daytime and nighttime maximum and minimum heat mitigation performance of PV canopy and fabric shading in *T*_mrt_, and UTCI.

Time	Scenario	Mean *T*_mrt_ (°C)			Mean UTCI (°C)		
		DSE	PV‐DSE	Fabric‐DSE	DSE	PV‐DSE	Fabric‐DSE
Day‐time	S1	66.8	−21.1	−25.2	41.5	−4.3	−4.5
	S2	65.6	−16.1	−21.1	43.2	−2.8	−3.2
	S3	69.1	−15.5	−20.1	44.7	−2.2	−2.8
	D‐Mean	67.2	−17.5	−22.1	43.1	−3.1	−3.5
Night‐time	S1	25.5	2.2	1.7	32.3	−0.7	−0.4
	S2	26.0	2.6	2.2	32.4	−0.4	0.0
	S3	37.6	0.1	−1.1	33.8	0.0	0.4
	N‐Mean	29.7	1.7	0.9	32.9	−0.4	0.0

*Note*: the maximum value(s) in each column is underlined and the minimum value(s) bold.

During the daytime, both shaded stations substantially reduced *T*_mrt_ and UTCI compared with the unshaded condition. The daytime mean *T*_mrt_ of the unshaded group was 67.2°C. PV canopy shading reduced *T*_mrt_ by 17.5°C on average, whereas fabric shading produced a larger reduction of 22.1°C. Across the three scenarios, the *T*_mrt_ reduction under PV canopy shading was 21.1°C in S1, 16.1°C in S2, and 15.5°C in S3. Under fabric shading, the corresponding reductions were 25.2, 21.1, and 20.1°C. Thus, fabric shading consistently exhibited stronger daytime radiant heat mitigation than PV canopy shading.

A similar pattern was observed for the UTCI. The daytime mean UTCI of the unshaded group was 43.1°C. PV canopy shading reduced UTCI by 3.1°C on average, whereas fabric shading reduced it by 3.5°C. In S1, the reductions were 4.3°C under PV canopy shading and 4.5°C under fabric shading. In S2, they were 2.8°C and 3.2°C, respectively, and in S3, they were 2.2°C and 2.8°C. These results indicate that both the shading stations improved daytime thermal comfort, with fabric shading showing a slightly stronger UTCI reduction. The reduction in both *T*_mrt_ and UTCI was largest in S1 and became smaller in the two extreme‐heat scenarios, indicating that shading remained effective under severe heat conditions, but its relative mitigation magnitude decreased as background heat stress intensified.

During the nighttime, the effects of the shading stations were much weaker and differed from those during the daytime. For *T*_mrt_, both shading stations showed a slight average nighttime warming effect. The nighttime mean *T*_mrt_ of the unshaded group was 29.7°C, whereas PV canopy shading increased *T*_mrt_ by 1.7°C on average, and fabric shading increased it by 0.9°C. The warming effect was most evident in S2, with increases of 2.6°C under PV canopy shading and 2.2°C under fabric shading. In S3, however, fabric shading reduced nighttime *T*_mrt_ by 1.1°C, which was different from the other nighttime cases.

The differences among the three groups for nighttime UTCI were small. The nighttime mean UTCI of the unshaded group was 32.9°C. PV canopy shading reduced UTCI by 0.4°C on average, whereas fabric shading showed no average change. In S1, both shading stations slightly reduced UTCI, with reductions of 0.7°C under PV canopy shading and 0.4°C under fabric shading. In S2, PV canopy shading reduced UTCI by 0.4°C, while fabric shading showed no difference. In S3, PV canopy shading was neutral, whereas fabric shading slightly increased UTCI by 0.4°C. Therefore, the nighttime UTCI effect was weak and scenario‐dependent.

Overall, the cooling performance of the two shading facilities was primarily observed during the daytime. Both PV canopy shading and fabric shading significantly reduced *T*_mrt_ and UTCI, but fabric shading performed better, especially in reducing *T*_mrt_. At night, the mitigation effect largely disappeared. Shading stations tended to slightly increase *T*_mrt_, particularly under PV canopy shading, whereas their influence on UTCI was minimal. Therefore, fabric shading showed a stronger daytime thermal comfort improvement, whereas PV canopy shading exhibited a more evident nighttime radiant warming tendency.4.5 Temporal variation of thermal stress in different shading scenarios

**FIGURE 15:**
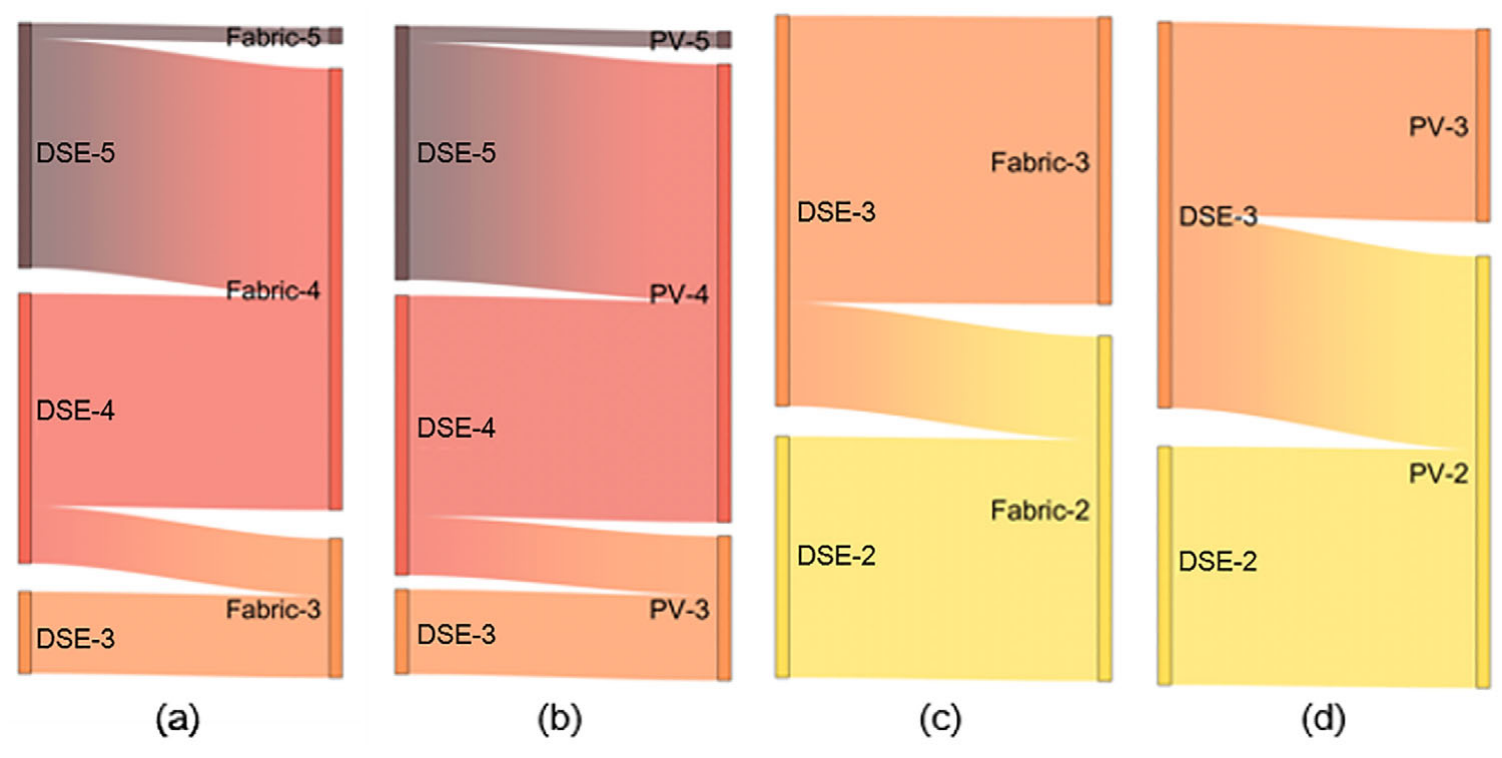
Transfer of thermal stress level (Levels 1–5): (a) Daytime fabric shading, (b) Daytime PV canopy shading, (c) Nighttime fabric shading, (d) Nighttime PV canopy shading.

Table 7 presents the distribution of UTCI‐related thermal stress levels under unshaded, PV canopy, and fabric shading during the daytime and nighttime periods. Figure 15 further illustrates the transfer of thermal stress levels from the unshaded condition to the two shaded conditions under the same conditions. The UTCI classification includes moderate, strong, very strong, and extreme heat stress. No thermal stress was observed in any group during the observation period.

**TABLE 7:** Thermal sensation/stress classification on UTCI scale.

Physiological stress	UTCI Range(°C)	Daytime (%)			Nighttime (%)			Whole day (%)		
		DSE	PV	Fabric	DSE	PV	Fabric	DSE	PV	Fabric
No thermal stress (Level 1)	+9 to +26	0	0	0	0	0	0	0	0	0
Moderate heat stress (Level 2)	+26 to +32	1.4	0	0	38.2	69.1	54.5	17.2	29.7	24.2
Strong heat stress (Level 3)	+32 to +38	15.1	23.3	23.3	61.8	30.9	45.5	35.1	26.6	51.6
Very strong heat stress (Level 4)	+38 to +46	45.2	74.0	74.0	0	0	0	25.8	42.2	22.7
Extreme heat stress (Level 5)	>+46	38.3	2.7	2.7	0	0	0	21.9	1.5	1.5

During the daytime, the unshaded condition showed the highest thermal stress level. Extreme heat stress accounted for 38.3% of the daytime period, whereas very strong heat stress accounted for 45.2%. Therefore, 83.5% of the daytime period under unshaded conditions was exposed to very strong or extreme heat stress. Under both PV canopy and fabric shading, the proportion of extreme heat stress was reduced sharply to 2.7%. Meanwhile, very strong heat stress accounted for 74.0%, and strong heat stress accounted for 23.3% under both shade conditions. This indicates that shading substantially reduced the occurrence of extreme heat stress, but most daytime periods under shade remained within the very strong heat stress category. Figure 15a,b further show that the daytime shading effect was mainly reflected in the transfer from higher stress levels to lower levels, especially from Level 5 to Level 4 or Level 3.

During nighttime, the severity of heat stress decreased markedly in all groups. Extreme and very strong heat stress were not observed at night. Under unshaded conditions, strong heat stress accounted for 61.8% of the nighttime period, whereas moderate heat stress accounted for 38.2%. Under PV canopy shading, the proportion of moderate heat stress increased to 69.1%, whereas that of strong heat stress decreased to 30.9%. Under fabric shading, moderate and strong heat stress accounted for 54.5% and 45.5% of the total stress, respectively. These results indicate that both shading stations reduced nighttime thermal stress levels, with PV canopy shading showing a larger shift from strong to moderate heat stress. This transfer pattern is also reflected in Figure 15c,d.

Over the whole day, the unshaded condition still showed a high proportion of severe thermal stress, with extreme heat stress accounting for 21.9% and very strong heat stress accounting for 25.8%. Under both shaded conditions, the whole‐day proportion of extreme heat stress decreased to 1.5%, indicating a clear reduction in the most severe thermal exposure for the bees. However, the distribution of the remaining stress levels differed between the two stations. Under PV canopy shading, very strong heat stress accounted for 42.2%, strong heat stress for 26.6%, and moderate heat stress for 29.7%. Under fabric shading, very strong heat stress was lower at 22.7%, while strong and moderate heat stress accounted for 51.6% and 24.2%, respectively. This suggests that fabric shading was more effective in reducing the duration of very strong heat stress over the whole day, whereas PV canopy shading showed a larger proportion of moderate heat stress.

Overall, both the shading stations effectively reduced the temporal duration of extreme heat stress. During the daytime, their main contribution was the reduction of Level 5 extreme heat stress, although very strong heat stress still dominated under shaded conditions. At night, the shading effect was mainly reflected in the shift from strong to moderate heat stress, particularly under PV canopy shading. Therefore, the two shading stations did not completely eliminate thermal stress, but they clearly reduced the occurrence of the most severe UTCI categories and redistributed thermal exposure toward lower‐stress levels.

### 4.5 Correlation Between UTCI and Microclimatic Variables

To identify the dominant microclimatic variables affecting UTCI during different daily periods, Table 8 presents the Pearson correlation coefficients between UTCI and five climatic variables, namely *T_a_*, *V_a_*, *RH*, *T_g_*, and *T*_mrt_, under DSE, PV, and fabric shading conditions. The analysis was conducted for four periods: early morning, midday/afternoon, late afternoon, and evening. The significance level of each correlation coefficient is indicated in Table 8. Unless otherwise specified as non‐significant, the reported correlations were statistically significant at *p* ≤ 0.001.

**TABLE 8:** Pearson linear correlation between UTCI and the climatic variables (*T_a_*, *V_a_*, *RH* and *T_g_, T*_mrt_) for each site and different period of the day during the period of measurements (2024.08.22 to 2024.08.24).

Period	Site	Ta	Va	RH	Tg	Tmrt
A Midday/Afternoon	DSE	r = 0.9051***	r = ‐0.8344***	r = ‐0.8673***	r = 0.9607***	r = 0.4505***
	PV canopy	r = 0.9141***	r = ‐0.6745***	r = ‐0.8525***	r = 0.9687***	r = 0.8253***
	Fabric	r = 0.9455***	r = ‐0.3892***	r = ‐0.8814***	r = 0.9839***	r = 0.8275***
B Late afternoon	DSE	r = 0.9016***	r = 0.7155***	r = ‐0.6364***	r = 0.9795***	r = 0.9818***
	PV canopy	r = 0.9389***	r = 0.3575**	r = ‐0.7309***	r = 0.9859***	r = 0.9811***
	Fabric	r = 0.9326***	r = 0.3330*	r = ‐0.7186***	r = 0.9877***	r = 0.9803***
C Night	DSE	r = 0.8636***	r = 0.2459**	r = ‐0.4978***	r = 0.9150***	r = 0.9201***
	PV canopy	r = 0.9032***	r = 0.1746*	r = ‐0.5809***	r = 0.8909***	r = 0.6399***
	Fabric	r = 0.9091***	r = ‐0.3090***	r = ‐0.6297***	r = 0.9271***	r = 0.8733***
D Early morning	DSE	r = 0.9719***	r = 0.8121***	r = ‐0.9015***	r = 0.9931***	r = 0.9808***
	PV canopy	r = 0.9423***	r = 0.5711***	r = ‐0.8840***	r = 0.9573***	r = 0.9581***
	Fabric	r = 0.9055***	r = ‐0.1987^ns^	r = ‐0.8352***	r = 0.9513***	r = 0.9151***

*Note*: *p ≤ 0.05, ** *p* ≤ 0.01, *** *p* ≤ 0.001, ^ns^ No significance.

During the early morning period, the UTCI was strongly correlated with most thermal variables under all three conditions. In the DSE group, *T_g_* and *T*_mrt_ showed the strongest correlations with UTCI, with correlation coefficients of 0.9931 and 0.9808, respectively. *T_a_* was also strongly correlated with UTCI (r = 0.9719), whereas *RH* showed a strong negative correlation (r = ‐0.9015). *V_a_* also had a strong positive correlation with UTCI in the DSE group (r = 0.8121). Under PV canopy shading, *T_g_*, *T*_mrt_, and *T_a_* remained strongly correlated with UTCI, with coefficients of 0.9573, 0.9581, and 0.9423, respectively. *RH* showed a strong negative correlation (r = ‐0.8840), whereas *V_a_* showed a moderate positive correlation (r = 0.5711, *p* ≤ 0.01). Under fabric shading, *T_g_*, *T*_mrt_, and *T_a_* were also strongly correlated with UTCI, with coefficients of 0.9513, 0.9151, and 0.9055, respectively. *RH* remained negatively correlated with UTCI (r = ‐0.8352), whereas *V_a_* showed no significant correlation (r = ‐0.1987, *p* > 0.05). These results indicate that the early morning UTCI was mainly associated with air temperature and radiant thermal variables, whereas the role of *V_a_* differed between the two shaded stations.

During the midday/afternoon period, *T_g_* and *T_a_* were the dominant variables associated with UTCI. In the DSE group, *T_g_* showed the strongest positive correlation with UTCI (r = 0.9607), followed by *T_a_* (r = 0.9051). *RH* showed a strong negative correlation (r = ‐0.8673), and *V_a_* was strongly correlated with UTCI (r = 0.8344). *T*_mrt_ showed a weaker but still significant positive correlation (r = 0.4505). Under PV canopy shading, *T_g_* and *T_a_* were strongly positively correlated with UTCI, with coefficients of 0.9687 and 0.9141, respectively. *RH* and *V_a_* were negatively correlated with UTCI, with coefficients of −0.8525 and −0.6745, respectively, whereas *T*_mrt_ showed a strong positive correlation (r = 0.8253). Under fabric shading, *T_g_* showed the strongest correlation with UTCI (r = 0.9839), followed by *T_a_* (r = 0.9455) and *T*_mrt_ (r = 0.8275), respectively. *RH* showed a strong negative correlation (r = ‐0.8814), whereas *V_a_* showed a weaker negative correlation (r = ‐0.3892). Therefore, during midday/afternoon, UTCI was mainly controlled by *T_g_* and *T_a_* under all three conditions, whereas *RH* showed an inverse relationship with UTCI, and *V_a_* exhibited different correlation directions between the unshaded and shaded environments.

During the late afternoon period, the correlations between UTCI and radiant thermal variables became particularly strong. In the DSE group, *T*_mrt_ and *T_g_* showed the strongest correlations with UTCI, with coefficients of 0.9818 and 0.9795, respectively. *T_a_* was also strongly correlated with UTCI (r = 0.9016), while *RH* showed a moderate negative correlation (r = ‐0.6364). *V_a_* was positive correlated with UTCI (r = 0.7155, *p* ≤ 0.01). Under PV canopy shading, *T_g_* and *T*_mrt_ remained strongly correlated with UTCI, with coefficients of 0.9859 and 0.9811, respectively, followed by *T_a_* (r = 0.9389). *RH* showed a strong negative correlation (r = −0.7309), whereas *V_a_* showed a weaker positive correlation (r = 0.3575, *p* ≤ 0.05). Under fabric shading, *T_g_*, *T*_mrt_, and *T_a_* were also strongly positively correlated with UTCI, with coefficients of 0.9877, 0.9803, and 0.9326, respectively. *RH* remained negatively correlated with UTCI (r = −0.7186), whereas *V_a_* showed a weak positive correlation (r = 0.3330, *p* ≤ 0.05). This indicates that, during the late afternoon, the UTCI was strongly associated with both radiant heat and air temperature under all three conditions, whereas the contribution of *V_a_* was relatively weaker.

During the nighttime period, UTCI still showed strong positive correlations with *T_a_* and *T_g_*, and *T*_mrt_. In the DSE group, *T*_mrt_ and *T_g_* were strongly correlated with UTCI, with coefficients of 0.9201 and 0.9150, respectively, followed by *T_a_* (r = 0.8636). *RH* showed a moderate negative correlation (r = −0.4978), whereas *V_a_* showed a weak positive correlation (r = 0.2459, *p* ≤ 0.05). Under PV canopy shading, *T_a_* and *T_g_* showed strong positive correlations with UTCI, with coefficients of 0.9032 and 0.8909, respectively. *T*_mrt_ also showed a positive correlation (r = 0.6390, *p* ≤ 0.01), while *RH* showed a negative correlation (r = −0.5809). *V_a_* was weakly correlated with UTCI (r = 0.1746; no significance). Under fabric shading, *T_g_*, *T_a_*, and *T*_mrt_ were strongly positively correlated with UTCI, with coefficients of 0.9271, 0.9091, and 0.8733, respectively. *RH* showed a negative correlation (r = −0.6297), while *V_a_* showed a weak negative correlation (r = −0.3090, *p* ≤ 0.01). These results suggest that the nighttime UTCI is mainly associated with *T_a_* and radiant thermal variables, whereas *V_a_* has a relatively weak and unstable relationship with the UTCI.

Overall, the correlation results showed that the UTCI was controlled by different combinations of variables throughout the day. *T_g_* consistently showed the strongest or near‐strongest positive correlation with UTCI in all periods and under all three conditions, indicating the central role of globe temperature‐related radiant heat exposure. *T_a_* was also strongly and positively correlated with UTCI throughout the day. *T*_mrt_ showed particularly strong correlations during the late afternoon, night, and early morning, but its correlation was weaker during the midday/afternoon period in the DSE group. *RH* was consistently negatively correlated with UTCI, reflecting the inverse variation between relative humidity and thermal stress during heating processes. *V_a_* showed the most variable behavior: it was positively correlated with UTCI in the DSE group, but its correlation became weaker, negative, or non‐significant under shaded conditions. This indicates that the UTCI variation under shading stations was governed mainly by air temperature and radiant thermal conditions, while the role of airflow was secondary and depended on shading type and time period.

To further examine the effects of weather conditions and underlying surface type, scenario‐specific correlation analyses were conducted for S1, S2, and S3 (Tables 9, 10, 11). Unless otherwise specified, the reported correlations were statistically significant at *p* ≤ 0.001.

**TABLE 9:** Pearson linear correlation between UTCI and the climatic variables (*T_a_*, *V_a_*, *RH* and *T_g_, T*_mrt_) for each site and different periods of the day during Scenario 1 (2024.08.22).

Period	Site	Ta	Va	RH	Tg	Tmrt
A Midday/Afternoon	DSE	0.9710***	−0.7045***	−0.9452***	0.9533***	−0.8237***
	PV canopy	0.7969***	−0.3423*	−0.7793***	0.9876***	0.8277***
	Fabric	0.8972***	−0.3178ns	−0.8012***	0.9873***	0.8311***
B Late afternoon	DSE	0.9663***	0.8439***	−0.9444***	0.9975***	0.9818***
	PV canopy	0.9816***	0.6389**	−0.9759***	0.9928***	0.9676***
	Fabric	0.9301***	0.4928*	−0.9367***	0.9867***	0.9460***
C Night	DSE	0.6946***	−0.2489^ns^	−0.3913*	0.6649***	0.3804*
	PV canopy	0.3326^ns^	−0.5306**	0.0243^ns^	0.8036***	0.4505**
	Fabric	0.7136***	−0.1516^ns^	−0.3936*	0.6862***	0.3766*

Note: ^*^ p ≤ 0.05, ^**^ p ≤ 0.01, ^***^ p ≤ 0.001, *^ns^ No significance*.

**TABLE 10:** Pearson linear correlation between UTCI and the climatic variables (*T_a_*, *V_a_*, *RH* and *T_g_, T*_mrt_) for each site and different periods of the day during Scenario 2 (2024.08.23).

Period	Site	Ta	Va	RH	Tg	Tmrt
A Midday/Afternoon	DSE	0.9268***	0.1765^ns^	−0.8761***	0.9915***	0.8179***
	PV canopy	0.8007***	−0.1055^ns^	−0.7624***	0.9912***	0.6444***
	Fabric	0.9314***	0.2231^ns^	−0.8907***	0.9446***	0.8287***
B Late afternoon	DSE	0.9940***	0.7445***	−0.9591***	0.9975***	0.9911***
	PV canopy	0.9846***	0.0562^ns^	−0.9436***	0.9934***	0.9673***
	Fabric	0.9897***	0.4500^ns^	−0.9522***	0.9964***	0.9885***
C Night	DSE	0.9895***	0.1955^ns^	−0.9726***	0.9882***	0.9212***
	PV canopy	0.9915***	0.1626^ns^	−0.9663***	0.9929***	0.9515***
	Fabric	0.9826***	−0.8195***	−0.9594***	0.9824***	0.9162***
D Early morning	DSE	0.9824***	0.9309***	−0.9871***	0.9991***	0.9819***
	PV canopy	0.9615***	0.6733***	−0.9623***	0.9770***	0.9680***
	Fabric	0.8822***	−0.6260**	−0.8992***	0.9907***	0.8775***

*Note*: ^*^ *p* ≤ 0.05, ^**^ *p* ≤ 0.01, ^***^ *p* ≤ 0.001, ^ns^ No significance.

**TABLE 11:** Pearson linear correlation between UTCI and the climatic variables (*T*_mrt_, *T_a_*, *V_a_*, *RH* and *T_g_*) for each site and different period of the day during Scenario 3 (2024.08.24).

Period	Site	Ta	Va	RH	Tg	Tmrt
A Midday/Afternoon	DSE	0.6443***	−0.0078^ns^	−0.4643**	0.9775***	0.7646***
	PV canopy	0.7389***	0.4334**	−0.7766***	0.9421***	0.9411***
	Fabric	0.6905***	0.4180**	−0.6248***	0.9708***	0.8850***
B Late afternoon	DSE	0.9796***	0.7538***	−0.8891***	0.9951***	0.9967***
	PV canopy	0.9742***	0.4647^ns^	−0.8889***	0.9974***	0.9921***
	Fabric	0.9743***	0.6233**	−0.8853***	0.9959***	0.9931***
C Night	DSE	0.8797***	0.4528***	−0.4675***	0.9223***	0.9545***
	PV canopy	0.9708***	0.3778**	−0.5559***	0.8943***	0.6275***
	Fabric	0.9307***	0.5842***	−0.6389***	0.9308***	0.9048***
D Early morning	DSE	0.9836***	0.8969***	−0.9842***	0.9994***	0.9836***
	PV canopy	0.9474***	0.6852***	−0.9546***	0.9801***	0.9544***
	Fabric	0.9515***	0.5902**	−0.9594***	0.9792***	0.9474***

*Note*: ^*^ *p *≤ 0.05, ^**^ *p* ≤ 0.01, ^***^ *p* ≤ 0.001, ^ns^ No significance

In S1, corresponding to the grassland scenario on a hot day, the UTCI was mainly associated with *T_a_*, *T_g_*, and *RH* during the daytime. *T_a_* and *T_g_* were strongly positively correlated with UTCI, whereas *RH* showed strong negative correlations. Compared with the other scenarios, the most distinct feature of S1 was the unstable role of *T*_mrt_ and *V_a_*. During midday/afternoon, *T*_mrt_ was negatively correlated with UTCI in the DSE group (r = ‐0.8237), but positively correlated with PV canopy shading and fabric shading. *V_a_* also showed weak or non‐significant relationships in some shaded cases, especially under fabric shading during the midday/afternoon (r = ‐0.3178, *p* > 0.05). At night, several correlations became weaker or non‐significant, indicating that the UTCI‐driving factors were less stable under relatively milder hot‐day conditions.

In S2, corresponding to the grassland scenario on an extreme heat day, the correlation structure became stronger and more consistent. *T_g_* showed very strong positive correlations with UTCI in almost all periods and groups, whereas *T_a_* and *T*_mrt_ also maintained high positive correlations. *RH* was consistently negatively correlated with UTCI, indicating a stable inverse relationship between humidity and heat stress under extreme heat. Compared with S1, the roles of the radiant and thermal variables became more prominent and persistent. In contrast, *V_a_* remained the most unstable variable. This was non‐significant in several cases and even showed a strong negative correlation under fabric shading at night (r = −0.8195), suggesting that airflow effects were more dependent on the local shading configuration than on the overall heat level.

In S3, corresponding to the high‐albedo surface scenario on an extreme heat day, UTCI showed a more radiation‐dominated correlation pattern. *T_g_* and *T*_mrt_ were strongly positively correlated with UTCI, whereas *RH* remained strongly negative. During midday/afternoon, the correlation between *T_a_* and UTCI was weaker than in S1 and S2, especially in the DSE group (r = 0.6443), whereas *T_g_* remained highly correlated with UTCI (r = 0.9775). This indicates that, over the high‐albedo surface, radiant heat exposure played a more dominant role than air temperature in determining UTCI. Therefore, compared with the grassland scenarios, S3 highlighted the enhanced influence of surface‐reflected radiation on outdoor thermal stress in the summer.

These findings are consistent with previous studies on outdoor thermal comfort and urban heat mitigation. Street‐scale interventions, such as building setbacks and roadside tree planting, have been shown to modify pedestrian‐level thermal exposure [22], whereas outdoor and semi‐outdoor thermal comfort in hot climates is strongly affected by the combined effects of radiation, air temperature, humidity, and airflow [23, 24]. Previous field studies have demonstrated that fabric shade structures can improve summer thermal comfort by reducing solar exposure [25], whereas photovoltaic and reflective shade surfaces may produce different sensible heat fluxes and pedestrian thermal responses because of their material properties [26]. Vegetation‐based shading has also been widely reported as an effective strategy for mitigating daytime urban heat stress [27]. From the perspective of thermal comfort assessment, Tmrt remains one of the most critical and challenging variables because it integrates complex shortwave and longwave radiation exchanges [28]. Therefore, combining field measurements with integrated urban climate assessment tools can further improve the evaluation of radiation‐driven thermal environments [29]. These studies support the present finding that the cooling performance of shading stations should be interpreted not only by air temperature reduction, but also by radiation control, airflow modification, material heating, and underlying‐surface conditions.

## 5 Conclusions

This study conducted a field‐based comparison of PV canopy and fabric shading under different weather and underlying surface scenarios. The main conclusions are as follows:

The heat mitigation effect of artificial shading was mainly concentrated during the daytime and was more evident in radiation‐related indicators than in air temperature. During daytime, PV canopy shading and fabric shading reduced mean *T_a_* by 1.0°C and 1.2°C, respectively, whereas the corresponding reductions in *T_g_* reached 3.5°C and 4.3°C. The differences between the shaded and unshaded groups became much weaker at night, with PV canopy shading showing almost no nighttime effect on *T_a_* and fabric shading producing only a slight warming effect of 0.2°C. The vertical profiles also showed that *T_a_* differences among different heights under the shaded stations were generally limited, whereas *T_g_* exhibited a more evident vertical difference between 1.10 m and 2.25 m. These results indicate that the main contribution of shading is to reduce daytime radiant heat exposure rather than substantially alter the air temperature field.

PV canopy and fabric shading improved the thermal environment through different mechanisms. During the full‐shading period, *T_g_* decreased from 47.2°C under the unshaded condition to 40.6°C under PV canopy shading and 39.4°C under fabric shading. Similarly, *T_a_* decreased from 39.6°C to 36.7°C and 36.9°C, respectively. Both shading stations substantially reduced the total radiation intensity, from 1865 W/m^2^ under direct sunlight to 1346 W/m^2^ under PV canopy shading and 1341 W/m^2^ under fabric shading. However, the PV canopy had a higher surface temperature than the fabric canopy and produced a larger increase in downward‐longwave radiation. As a result, fabric shading showed stronger daytime radiant‐heat mitigation, with average reductions of 22.1°C in *T*_mrt_ and 3.5°C in UTCI, compared with 17.5°C and 3.1°C under PV canopy shading. Therefore, fabric shading was more effective in reducing radiant heat exposure, while PV canopy shading had the additional potential advantage of maintaining airflow and providing energy‐related functions, but with a stronger canopy‐surface heating effect.

The performance of shading facilities was strongly constrained by weather conditions and the underlying surface. In the grassland scenarios, both shading stations reduced daytime ground surface temperature. PV canopy shading and fabric shading reduced mean *ST* by 2.8°C and 1.4°C in S1, and by 1.2°C and 0.4°C in S2, respectively. However, over the high‐albedo surface in S3, ST increased by 1.8°C under PV canopy shading and 0.9°C under fabric shading. This indicates that the thermal response of the ground surface under shading was not uniform but depended on the surface material and background heat intensity. The correlation analysis further showed that, under the high‐albedo surface scenario, UTCI was strongly associated with radiant thermal variables such as *T_g_* and *T*_mrt_, suggesting that reflected and accumulated radiant heat played a dominant role in influencing outdoor heat stress.

Both shading stations reduced the duration and intensity of thermal stress but did not completely eliminate the heat risk under extreme heat conditions. During the daytime, the unshaded group was exposed to extreme heat stress for 38.3% of the period, whereas this proportion decreased to 2.7% under both PV canopy and fabric shading. Over the entire day, the proportion of extreme heat stress decreased from 21.9% under unshaded conditions to 1.5% under both shaded conditions. However, very strong heat stress still occurred for a considerable proportion of the daytime under shaded conditions, indicating that shading mainly reduced the highest heat‐stress category rather than fully removing thermal stress from the environment. Correlation analysis showed that UTCI was mainly governed by *T_a_*, *T_g_*, *T*_mrt_, and *RH*, whereas the role of *V_a_* was weaker and more variable among the different types of shade and time periods.

Overall, the findings suggest that artificial shading should be selected based on local environmental conditions and design objectives. Fabric shading is more suitable when the primary goal is radiant heat mitigation, while PV canopy shading is more appropriate for open and well‐ventilated spaces where solar‐energy utilization and airflow maintenance are also considered. In hot outdoor spaces, shading design should be combined with ground surface material control and ventilation planning to achieve more effective heat mitigation.

6

**Nomenclature**
DSE: Direct solar exposure
L↓: Downward longwave radiation
L↑: Upward longwave radiation from the ground
OTC: Outdoor thermal comfort
PV: Photovoltaic
*RH*
: Relative humidity
S↓: Downward shortwave radiation
S↑: Upward shortwave radiation
*ST*
: Surface temperature at the ground level
*T_g_*
: Black globe temperature
*T*_mrt_
: Mean radiant temperature
*T_s_*
: Surface temperature of shading canopy
UTCI: Universal Thermal Climate Index
UHI: Urban heat island
*V*_a_
: Wind speed

## Author Contributions

Liyue Jin: Writing the original draft, Software, Methodology, Investigation, Formal analysis, Data curation, Conceptualization. Xin Dong: Methodology, Investigation, Formal analysis. Deo Prasad: Writing, review & editing, Investigation, Formal analysis, Supervision. Bao‐Jie He: Writing, review & editing, Investigation, Formal analysis, Supervision, Conceptualization.

## Conflicts of Interest

The authors declare no conflicts of interest.

## Data Availability

The data that support the findings of this study are available from the corresponding author upon reasonable request.

## References

[1] B. J. He , J. Wang , H. Liu , and G. Ulpiani , “Localized Synergies Between Heat Waves and Urban Heat Islands: Implications on human Thermal Comfort and Urban Heat Management,” Environmental Research 193 (2021): 110584, 10.1016/j.envres.2020.110584. 33285157

[2] C. Mora , B. Dousset , I. R. Caldwell , et al., “Global Risk of Deadly Heat,” Nature Climate Change 7, no. 7 (2017): 501–506, 10.1038/nclimate3322.

[3] V. K. Turner , A. Middel , and J. K. Vanos , “Shade Is an Essential Solution for Hotter Cities,” Nature 619, no. 7971 (2023): 694–697, 10.1038/d41586-023-02311-3. 37495873

[4] R. Emmanuel , An Urban Approach to Climate Sensitive Design: Strategies for the Tropics (Taylor & Francis, 2012), 10.4324/9780203414644.

[5] A. M. R. Nishimwe and S. Reiter , “Towards Nearly Zero‐Energy Residential Neighbourhoods in the European Union: A Case Study,” Renewable and Sustainable Energy Reviews 135 (2021): 110198.

[6] J. Yang , Y. Liang , Z. Zhong , K. Dharmasastha , Y. Xie , and J. L. Niu , “Thermal Comfort Investigation of Membrane‐assisted Radiant Cooling in Outdoor Settings,” Sustainable Cities and Society 113 (2024), 105634.

[7] H. Swaid , “Intelligent Urban Forms (IUF) a New Climate‐Concerned, Urban Planning Strategy,” Theoretical and Applied Climatology 46 (1992): 179–191, 10.1007/BF00866098.

[8] M. W. Yahia and E. Johansson , “Landscape Interventions in Improving Thermal Comfort in the Hot Dry City of Damascus, Syria—The Example of Residential Spaces With Detached Buildings,” Landscape and Urban Planning 125 (2014): 1–16, 10.1016/j.landurbplan.2014.01.014.

[9] D. Elgheznawy and S. Eltarabily , “The Impact of Sun Sail‐Shading Strategy on the Thermal Comfort in School Courtyards,” Building and Environment 202 (2021): 108046, 10.1016/j.buildenv.2021.108046.

[10] N. Kántor , Á. Gulyás , and C. Gál , “Relevance of Urban Trees and Sun Shades Regarding Summertime Heat Stress Reduction: Field Surveys From Pécs, Hungary,” in St International Congress of Biometeorology (2017).

[11] L. Zhang , D. Wei , Y. Hou , et al., “Outdoor Thermal Comfort of Urban Park—A Case Study,” Sustainability 12, no. 5 (2020): 1961.

[12] H. Ikeda , T. Nakaya , A. Nakagawa , and Y. Maeda , “An investigation of Indoor Thermal Environment in Semi‐Cold Region in Japan—Validity of Thermal Predictive Indices in Nagano During the Summer Season,” Journal of Building Engineering 35 (2021): 101897, 10.1016/j.jobe.2020.101897.

[13] J. Zhang , P. Cui , and H. Song , “Impact of Urban Morphology on Outdoor Air Temperature and Microclimate Optimization Strategy Base on Pareto Optimality in Northeast China,” Building and Environment 180 (2020): 107035, 10.1016/j.buildenv.2020.107035.

[14] P. K. Cheung and C. Y. Jim , “Comparing the Cooling Effects of a Tree and a Concrete Shelter Using PET and UTCI,” Building and Environment 130 (2018): 49–61, 10.1016/j.buildenv.2017.12.013.

[15] M. C. Peel , B. L. Finlayson , and T. A. McMahon , “Updated World Map of the Köppen‐Geiger Climate Classification,” Hydrology and Earth System Sciences 11, no. 5 (2007): 1633–1644, 10.5194/hess-11-1633-2007.

[16] Y. Nakamura , Y. Asano , A. Suzuki‐Parker , and H. Kusaka , “Verification of Heat Stress Mitigation Effects by UV Parasols Using UTCI Observations and Thermal Sensory Questionnaire Survey,” Building and Environment 266 (2024): 112025, 10.1016/j.buildenv.2024.112025.

[17] H. Lee and H. Mayer , “Solar Elevation Impact on the Heat Stress Mitigation of Pedestrians on Tree‐lined Sidewalks of EW Street Canyons‐ Analysis Under Central European Heat Wave Conditions,” Urban Forestry & Urban Greening 58 (2021): 126905.

[18] S. Jia , Y. Wang , N. H. Wong , W. Chen , and X. Ding , “Influences of the Thermal Environment on Pedestrians' Thermal Perception and Travel Behavior in Hot Weather,” Building and Environment 226 (2022): 109687, 10.1016/j.buildenv.2022.109687.

[19] International Organization for Standardization , Ergonomics of the Thermal Environment—Instruments for Measuring Physical Quantities (1998).

[20] O. Potchter , P. Cohen , T. P. Lin , and A. Matzarakis , “Outdoor human Thermal Perception in Various Climates: A Comprehensive Review of Approaches, Methods and Quantification,” Science of the Total Environment 631 (2018): 390–406, 10.1016/j.scitotenv.2018.02.276. 29525717

[21] P. Bröde , D. Fiala , K. Błażejczyk , et al., “Deriving the Operational Procedure for the Universal Thermal Climate Index (UTCI),” International Journal of Biometeorology 56 (2012): 481–494. 21626294 10.1007/s00484-011-0454-1

[22] Z. Tan , A. Wang , T. E. Morakinyo , E. H. K. Yung , and E. H. W. Chan , “Assessing the Mitigation Performance of Building Setback From Street and the Combination With Roadside Tree Planting,” Building and Environment 212 (2022): 108814.

[23] J. A. Acero , L. A. Ruefenacht , E. J. Koh , Y. S. Tan , and L. K. Norford , “Measuring and Comparing Thermal Comfort in Outdoor and Semi‐outdoor Spaces in Tropical Singapore,” Urban Climate 42 (2022): 101122.

[24] E. Ng and V. Cheng , “Urban Human Thermal Comfort in Hot and Humid Hong Kong,” Energy and Buildings 55 (2012): 51–65, 10.1016/j.enbuild.2011.09.025.

[25] T. Sung , H. Tanaka , and Y. Ohashi , “Evaluation of the Effects of Fabric Shade Facilities on Outdoor Thermal Comfort in Summer: A Case Study in Tokyo,” Building and Environment 180 (2020): 107039.

[26] J. V. Pham , A. Baniassadi , K. E. Brown , J. Heusinger , and D. J. Sailor , “Comparing Photovoltaic and Reflective Shade Surfaces in the Urban Environment: Effects on Surface Sensible Heat Flux and Pedestrian Thermal Comfort,” Urban Climate 29 (2019): 100500, 10.1016/j.uclim.2019.100500.

[27] Z. Tan , K. K. Lau , and E. Ng , “Urban Tree Design Approaches for Mitigating Daytime Urban Heat Island Effects in a High‐Density Urban Environment,” Energy and Buildings 114 (2016): 265–274, 10.1016/j.enbuild.2015.06.031.

[28] N. Kántor and J. Unger , “The Most Problematic Variable in the Course of Human‐Biometeorological Comfort Assessment—The Mean Radiant Temperature,” Central European Journal of Geosciences 3, no. 1 (2011): 90–100.

[29] F. Lindberg , C. S. B. Grimmond , A. Gabey , et al., “Urban Multi‐Scale Environmental Predictor (UMEP): An Integrated Tool for City‐Based Climate Services,” Environmental Modelling & Software 99 (2018): 70–87, 10.1016/j.envsoft.2017.09.020.

